# eEF2K is a poor prognostic factor and novel molecular target in pancreatic cancer: regulating tumor growth and progression via the tumor microenvironment

**DOI:** 10.1038/s41419-025-07803-w

**Published:** 2025-07-07

**Authors:** Didem Karakas, Ahmed Ashour, Hamada Ahmed Mokhlis, Nermin Kahraman, Recep Bayraktar, Sayra Dilmac, Nashwa N. Kabil, Mumin Alper Erdogan, Egemen Dere, Engin Ulukaya, Bulent Ozpolat

**Affiliations:** 1 Department of Experimental Therapeutics, The University of Texas-MD Anderson Cancer Center, Houston, TX USA; 2 Department of Biology, Faculty of Arts and Sciences, Uludag University, Bursa, Turkey; 3 Department of Medical Biotechnology, Graduate School of Health Sciences, Acibadem Mehmet Ali Aydinlar University, Istanbul, Turkey; 4 Department of Pharmacology and Toxicology, Faculty of Pharmacy, Al-Azhar University, Cairo, Egypt; 5 Houston Methodist Research Institute, Houston, TX USA; 6 Department of Physiology, Faculty of Medicine, Izmir Katip Celebi University, Izmir, Turkey; 7 Department of Clinical Biochemistry, Faculty of Medicine, Istinye University, Istanbul, Turkey; 8 Methodist Neal Cancer Center, Houston, TX USA

**Keywords:** Cancer microenvironment, RNAi

## Abstract

Pancreatic ductal adenocarcinoma (PDAC) is one of the most lethal cancers, with an average survival time of only six months following diagnosis, even with currently available therapies. Thus, PDAC represents a significant therapeutic challenge, necessitating a deeper understanding of its biology and tumor microenvironment (TME) to develop more effective treatments and improve patient outcomes. Here, we report that the expression of Eukaryotic Elongation Factor-2 Kinase (eEF2K) is associated with shorter patient survival and demonstrate that eEF2K signaling is critical for the PDAC tumor growth and regulated by the TME. Furthermore, in vivo targeted genetic inhibition of eEF2K suppressed tumor growth in two different PDAC mouse models, reduced tumor-associated macrophages (TAMs), and induced marked apoptosis in tumor tissues without any signs of toxicity. Our data suggest that eEF2K knockdown diminishes the activity of the AXL receptor tyrosine kinase and reduces the expression of macrophage-derived factors, such as Monocyte Chemoattractant Protein-1 (MCP1), along with the Gas6/AXL signaling pathway in PDAC cells. Additionally, analysis of the NCI-TCGA PDAC patient database further showed that eEF2K expression, in the presence of TAM markers, correlates with even shorter patient survival. TAM-released factors, such as MCP1, Gas6, and exosomes, induce eEF2K expression in PDAC cells, as well as the activity of AXL, SRC, VEGF, Snail, and MMP2, contributing to epithelial-to-mesenchymal transition (EMT), invasion, metastasis, and angiogenesis. In conclusion, our findings reveal for the first time that eEF2K is a critical oncogenic driver of PDAC tumor growth and thus targeting eEF2K represents a promising and novel therapeutic strategy for PDAC.

## Introduction

Pancreatic ductal adenocarcinoma (PDAC), accounting for 90% of pancreatic cancer cases, is among the most lethal malignancies, with a median survival time of only six months post-diagnosis [1]. Despite advancements in science and technology, the overall 5-year survival rate for metastatic PDAC patients remains alarmingly low, at less than 10% [2]. The majority ( ~ 85%) of patients are diagnosed with either locally advanced or metastatic disease, and current therapies have had minimal impact on survival. In the metastatic setting, treatments such as FOLFIRINOX (folinic acid, 5-fluorouracil, irinotecan, and oxaliplatin) and nab-paclitaxel plus gemcitabine have achieved only modest improvements in survival [3]. Furthermore, immunotherapy and targeted therapies have not significantly improved the prognosis of PDAC patients, highlighting the urgent need for the development of more effective treatments.

A key factor contributing to the aggressive and incurable nature of PDAC is its unique tumor microenvironment (TME) [4]. Accumulating evidence suggests that pancreatic cancer TME plays a critical role in treatment failures and promotes tumor growth and progression [5–9]. Recent studies have shown that the TME contributes to drug resistance, metastasis, angiogenesis, and the evasion of apoptosis in solid cancers, including PDAC [10–14]. Tumor-associated macrophages (TAMs), which are abundant immune cells within the TME [15], are negatively correlated with prognosis in PDAC [16–18]. Macrophages within the TME undergo reprogramming into either M1 “anti-tumorigenic” or M2 “pro-tumorigenic” TAMs in response to signals from tumor cells and the surrounding microenvironment [19, 20]. The interactions between cancer cells and TAMs significantly influence the behavior and function of both cell types, as well as influencing other cell types within the TME. While cancer cells secrete soluble factors and exosomes that polarize M1 macrophages into the M2 phenotype, M2 macrophages, in turn, release cytokines and chemokines that promote cancer cell growth and metastasis [21–23]. However, the specific mediators and mechanisms underlying the crosstalk between PDAC cells and TAMs remain poorly understood. Monocyte chemoattractant protein-1 (MCP-1/CCL2) is one of the most critical chemokines regulating the recruitment and infiltration of monocytes into tumors [24]. MCP-1/CCR2 signaling promotes the differentiation of monocytes into macrophages [25, 26], and drives cancer cell migration and invasion [27]. In pancreatic cancer, MCP-1 expression is associated with poor prognosis and has been identified as a potential therapeutic target [28]. Furthermore, circulating levels of MCP-1 in the serum of pancreatic cancer patients correlate with macrophage infiltration into tumors [29, 30], suggesting that both MCP-1 and TAMs may represent critical therapeutic targets in PDAC.

Eukaryotic elongation factor-2 kinase (eEF2K) is a calcium/calmodulin-dependent enzyme that negatively regulates protein synthesis [31, 32]. Under stress conditions, eEF2K is activated to slow down protein synthesis, conserving energy and promoting cell survival [33]. The TME is characterized by metabolic challenges, including hypoxia, nutrient depletion, and the accumulation of waste products. eEF2K has been shown to facilitate cancer cell survival under these conditions [34, 35]. Additionally, eEF2K promotes cancer cell survival, proliferation, and chemoresistance through mechanisms independent of translational control, including the activation of oncogenic signaling pathways such as c-Myc, cyclin D1, integrin β1, IGFR1B, PDL1, PP2A-A, and the PI3K/AKT/mTOR, STAT3, and SRC/FAK pathways [36–40]. However, the role of eEF2K in PDAC tumorigenesis remains poorly understood.

Given the multifaceted oncogenic role of eEF2K in solid tumors, we investigated its role in pancreatic cancer tumorigenesis and the TME. Our findings revealed that eEF2K serves as a significant marker for poor patient survival and is highly upregulated in PDAC cells and promotes tumor growth. In vivo targeting of eEF2K suppressed tumor growth, induced marked apoptosis and reduced the accumulation of TAMs in PDAC tumors, with no observed toxicity. Additionally, we found that TAM-derived factors, including MCP-1, Gas6, and exosomes, induce eEF2K expression in PDAC cells. Our study suggests that eEF2K mediates a complex intratumoral communication network between PDAC cells and macrophages, thereby driving tumor growth and progression. Therefore, targeting the eEF2K/TAM axis may offer a novel therapeutic strategy to improve the survival outcomes of pancreatic cancer patients.

## Materials and methods

### Cell lines and cell culture conditions

Human pancreatic cancer cell lines (PANC-1 and MiaPaCa-2) and human acute monocytic leukemia cell line (THP-1) were sourced from the American Type Culture Collection (ATCC) (Manassas, VA, USA). Human pancreatic stellate cells (PSCs) were provided by Dr. Rosa Hwank (MD Anderson Cancer Center). PANC-1, MiaPaCa-2, and PSC cells were cultured in Dulbecco’s Modified Eagle’s Medium (DMEM)/F12 supplemented with 10% fetal bovine serum (FBS) and 100 U/ml penicillin-streptomycin (P/S) solution. THP-1 cells were maintained in Roswell Park Memorial Institute (RPMI)-1640 supplemented with 10% FBS and 100 U/ml P/S solution. All cells were grown in 25 cm^2^ or 75 cm^2^ cell culture flasks at 37 °C in a humidified atmosphere containing 5% CO_2_ and regularly tested for mycoplasma contamination.

### siRNA transfection

PANC-1 or MiaPaCa-2 cells were seeded in 6-well plates at densities of 2.5 × 10^5^ and 3.5 × 10^5^ cells per well, respectively. After 24 hours of incubation, cells were transiently transfected with 50 or 100 nM concentrations of control siRNA or eEF2K siRNA (Sigma-Aldrich, St. Louis, MO, USA) using Hiperfect transfection reagent (Qiagen, Hilden, Germany) in Opti-MEM Reduced Serum Medium (Life Technologies, Carlsbad, CA, USA), according to the manufacturer’s protocol. Six hours post-transfection, Opti-MEM medium was replaced with culture medium containing 10% FBS, and cells were incubated for an additional 48 hours before further experimentation.

### Overexpression of eEF2K using lentiviral vector

To achieve stable eEF2K overexpression, PANC-1 cells were infected with lentiviral plasmids containing the eEF2K coding sequence (NM_013302.3) under the CMV promoter (LPP-U0633-Lv105) or mock vector (LPP-NEG-Lv103), following the manufacturer’s protocol. Initially, PANC-1 cells were seeded into a 96-well plate (1 × 10^3^ cells/well) and incubated overnight to allow for cell attachment. The next day, lentiviral particles were diluted in cell culture media containing 5% FBS and 1% P/S, supplemented with polybrene (EMD Millipore Corporation, Billerica, MA, USA) at a final concentration of 8 μg/ml, and added to the wells. After 48 hours, the media was replaced with puromycin-containing media (10 μg/ml) for selection over three weeks (Invitrogen/Life Technologies, Carlsbad, CA). Overexpression of eEF2K was confirmed by Western blot analysis.

### Polarization of monocytes into macrophages

THP-1 monocytic cells were seeded in 75 cm^2^ flasks at a density of 2–2.5 × 10^6^ cells per flask. To induce differentiation into mature macrophages (M0 type), cells were treated with 10 ng/ml Phorbol 12-myristate 13-acetate (PMA) (Sigma-Aldrich, St. Louis, MO, USA) for 24 hours. Subsequently, PMA-containing media was gently aspirated, and fresh media was added (without PMA). For M2 macrophage polarization, IL-4 and IL-13 were added at a final concentration of 25 ng/ml, and cells were incubated for an additional 24 hours. The levels of CD68, CD163, and CD206 were assessed by Western blot to confirm polarization.

### Indirect co-culture experiments

PANC-1, MiaPaCa-2 and PSC cells were seeded in 6-well plates at a density of 3 × 10^5^ cells/well. Cells were co-cultured with THP-1 monocytes or M1/M2 macrophages using a transwell insert system (24 mm diameter, 0.4 µm pore size, Corning, New York, NY, USA) for 48 hours. After co-culture, the inserts were removed, and cells were subjected to further analyses.

### Conditioned media collection

THP-1 cells were cultured in 75 cm^2^ flasks and polarized into macrophages, as described above. The media were replaced with media containing 1% FBS, and cells were incubated for an additional 24 hours. The media were then collected, centrifuged at 1000 rpm for 5 minutes, filtered through 0.22 μm syringe filters, and used immediately. PANC-1, MiaPaCa-2, and PSC cells were treated with conditioned media mixed with fresh media (1:1) for 48 hours.

### Exosome isolation

THP-1 cells were seeded into 75 cm^2^ flasks, with two flasks used per cell type (THP-1 cells, M0 macrophages, and M2 macrophages; six flasks total). Cells were differentiated as previously described, and the media was replaced with RPMI-1640 supplemented with 10% exosome-depleted FBS (System Biosciences, Palo Alto, CA, USA). After 24 hours of incubation with exosome-depleted media, supernatants were collected, and cell debris removed by centrifugation at 1000 x g for 5 minutes at +4 °C, followed by additional centrifugation at 2000 x g for 30 minutes at +4 °C. Using an ultracentrifuge (Beckman Coulter, Brea, CA, USA), supernatants were centrifuged at 60,000 x g for 45 minutes at +4 °C and then at 240,000 x g for 90 minutes. The exosome pellet was resuspended in filtered-phosphate-buffered saline (PBS), quantified using NanoSight (Malvern, UK), and used immediately.

### Measurement of secreted MCP-1 levels

Following the indirect co-culture of PANC-1 cells with THP-1 cells/macrophages for 48 hours, culture media were collected from each well of the 6-well plates. Media from the THP-1 cells, M0 and M2 macrophages, and PANC-1 cells cultured alone (without co-culture) were also collected as controls. MCP-1 concentrations were measured using a human specific MCP-1 ELISA kit (R&D Systems, Minneapolis, MN, USA) according to manufacturer’s instructions.

### Recombinant MCP-1 and Gas6 treatments

Recombinant human MCP-1/CCL2 protein (R&D Systems, Minneapolis, MN, USA) was dissolved in sterile PBS (with 0.1% bovine serum albumin) at 100 μg/ml as stock solution. Recombinant human Gas6 protein (R&D Systems, Minneapolis, MN, USA) was dissolved in sterile water at a stock concentration of 500 μg/ml. PANC-1 cells were seeded into 6-well plates at a density of 1.5 × 10^5^ cells per well and incubated overnight to allow attachment. For Gas6 administration, the media were replaced with FBS-free media, and cells were incubated overnight. Cells were then treated with varying concentrations of Gas6 (200, 400, 600 ng/ml) for 30 minutes. For MCP-1 treatment, cells were exposed to different concentrations of MCP-1 (10, 25, 50 ng/ml) in FBS-free media for 24 hours. Subsequently, cells were stimulated with MCP-1 and Gas6, as described, and then used for further experiments.

### Proliferation and colony formation assay

The colony-forming ability of PANC-1 cells was assessed by clonogenic assay. PANC-1 cells were plated in 6-well plates at a density of 5 × 10^2^ cells/well. After overnight incubation, cells were treated with exosomes (1.5 × 10^8^ exosomes/well) or siRNAs (control and eEF2K, 25 nM) for 24 hours. The media were then replaced with fresh media, and cells were cultured for 10–14 days until visible colonies formed. Colonies were stained with crystal violet (0.5% w/v) and counted using ImageJ software (National Institutes of Health, Bethesda, MD, USA).

### Cell motility/migration assay

Cell migration was measured using the wound healing assay. PANC-1 cells were seeded at a density of 3 × 10^5^ cells/well in 6-well plates and incubated for 24 hours. Wounds were created using a 200-μl sterile pipette tip, and cells were gently washed with culture media to remove cell debris. Cells were then treated with exosomes (1.5 × 10^8^), siRNAs (control and eEF2K, 50 nM), or co-cultured with monocytes/macrophages for 48 hours. Immediately after scratching, the cells were photographed using a phase-contrast microscope (Nikon Eclipse TE-200-U) to measure the wound width at 0 hours. Additional photographs were taken at 24 and 48 hours post-wounding. At least three random non-overlapping images were captured from each well.

### Cell invasion assay

PANC-1 cells were plated in 6-well plates and subjected to the same treatments (exosome, siRNA, or co-culture) as described previously. After 24 hours, cells were collected, washed with serum-free media, and resuspended in 200 µl of serum-free culture media (5 × 10^4^) and added onto 24-well plate Transwell inserts (8-μm-pore size; Fisher Scientific) coated with Matrigel basement membrane (0,7 mg/mL; BD Biosciences). The lower chambers were filled with 500 µl of media containing 10% FBS as a chemoattractant. 24 hours later, the invading cells were fixed, stained with a Hema 3 staining kit (Thermo Scientific, Waltham, MA, USA), and the cells in the upper chamber were removed by wiping with a cotton swab and then the membranes were photographed. The number of cells that had invaded to the lower side of the filter was counted in at least 6 fields using ImageJ software and the results expressed as the mean number of cells from triplicate measurements.

### Western blot

Following the abovementioned treatments, the cells were washed twice in ice-cold PBS, and lysed in 1X RIPA buffer (Thermo Scientific, Waltham, MA, USA) containing protease and phosphatase inhibitors for 30 minutes at +4 °C. Lysates were centrifuged at 13,000 x g for 10 minutes at +4 °C, and supernatants were collected. Total protein concentration was measured using the Pierce BCA protein assay kit (Thermo-Fisher Scientific, Waltham, MA, USA). Then, lysates were resuspended in Laemmli loading buffer (Bio-Rad, Hercules, CA, USA) and heated at 95 °C for 5 minutes. Equal amounts of protein (30–40 µg protein/lane) were subjected to SDS-PAGE with a 4% to 15% gradient for protein separation and electrotransferred to polyvinylidene difluoride (PVDF) membranes. The membranes were blocked with 5% dry milk in TBS containing 0.1% Tween-20 (TBS-T) and then probed with the following primary antibodies: eEF2K, p-eEF2 (Thr56), Src, p-Src (Tyr416), MMP-2, Snail, CCR2, (Cell Signaling Technology, Danvers, MA, USA); CD68, CD163, CD206 (Abcam); MCP-1, Axl, p-Axl (Tyr702) (R&D Systems, Minneapolis, MN, USA); Gas6 (Abcam, Cambridge, UK). The membranes were then labeled with horseradish peroxidase-conjugated anti-rabbit, anti-mouse, or anti-goat secondary antibodies (Cell Signaling Technology, Danvers, MA, USA). Chemiluminescent detection was performed with HyGLO Chemiluminescent HRP Antibody Detection Reagent (Denville Scientific). Each membrane was exposed to film (Kodak) in a dark room for 0.5–10 min. For subsequent detections with different antibodies, membranes were stripped using Restore™ PLUS Western Blot Stripping Buffer (Thermo Scientific, Waltham, MA, USA) for 15 min. β-actin (Sigma-Aldrich, St. Louis, MO, USA) and GAPDH (Cell Signaling Technology, Danvers, MA, USA) were used as loading control.

### Reverse phase protein array (RPPA)

Briefly, PANC-1 cells were seeded and then treated with exosomes derived from THP-1 cells, M0, and M2 macrophages for 48 h. The cells were washed twice with PBS, and then 100 μl of lysis buffer-containing 1% Triton X-100, 50 mM HEPES (pH 7.4), 150 mM NaCl, 1.5 mM MgCl_2_, 1 mM EDTA, 100 mM NaF, 10 mM sodium pyrophosphate, 1 mM Na_3_VO_4_, 10% glycerol, and protease and phosphatase inhibitors (Roche Applied Science, Penzberg, Germany) were added to the plate on ice. Cells were scraped and centrifuged at 14,000 rpm for 10 min at +4 °C. Supernatants were collected and total proteins were quantified using Pierce BCA protein assay kit (Thermo-Fisher Scientific, Waltham, MA, USA). The concentration of proteins was adjusted to 1.5 μg/μl. Then, 4x SDS sample buffer (40% glycerol, 8% SDS, 0.25 M Tris-HCl, 10% 2-mercaptoethanol, pH 6.8) was added to cell lysates. Protein samples were denatured and stored at −80 °C until RPPA processing. RPPA analysis was performed at the Functional Proteomics RPPA Core Facility of The University of Texas MD Anderson Cancer Center.

### PDAC tumor xenograft models

Athymic female nu/nu mice (BALB/c nude) (5 weeks old) were obtained from the Department of Experimental Radiation Oncology at MD Anderson Cancer Center, Houston, TX. All in vivo studies were conducted according to an experimental protocol approved by the MD Anderson Institutional Animal Care and Use Committee. PANC-1 or MiaPaCa-2 cells (2 × 10^6^ cells) were injected into the left flank of each mouse. Two weeks after injection, when tumor size reached about 5 mm, each mouse was treated with liposomal siRNAs (control or eEF2K). All siRNAs for in vivo delivery were incorporated into nanoliposomes (NL) composed of 1,2-dioleoyl-sn-glycero-3- phosphatidylcholine as previously described [36]. Each mouse received 0.3 mg/kg (*n* = 5) non-silencing control siRNA or eEF2K siRNA twice a week (i.v. injection into the tail vein in 100 µL saline) for four weeks. After completion of treatments, mice were euthanized with CO_2_, and tumor tissues were collected for Western blot and immunohistochemistry analysis.

### Immunohistochemistry (IHC)

Tumor tissues were fixed and embedded in paraffin. Tissue sections (5 μm) were stained with hematoxylin and eosin. Ki-67 and CD31 antibodies were used for immunohistochemical evaluation of cell proliferation and angiogenesis, respectively. Stainings were performed according to the manufacturer’s protocol. F4/80 antibody was used to stain tumor-infiltrated macrophages. The slides were developed with 3,3’-Diaminobenzidine (DAB) substrate (Vector Laboratories Inc.) and counterstained with hematoxylin solution. The slides were analyzed by microscopy (Nikon Eclipse TE-200-U; Nikon Instruments, Inc., Melville, NY, USA) and positive cells were counted in at least 5 random fields per slide.

### Statistical analyses

Data were expressed as means or fold changes ± standard deviations (SDs). Statistical analysis was performed using the Student’s t-test and one-way ANOVA to determine statistical significance by using GraphPad Prism 9.0 statistical software for Windows. *P* values indicate the probability that the means compared are equal with *P < 0.05, **P < 0.01, ***P < 0.001, ****P < 0.0001.

## Results

### eEF2K expression is upregulated in PDAC cells and associated with poor patient survival

**Fig. 1:**
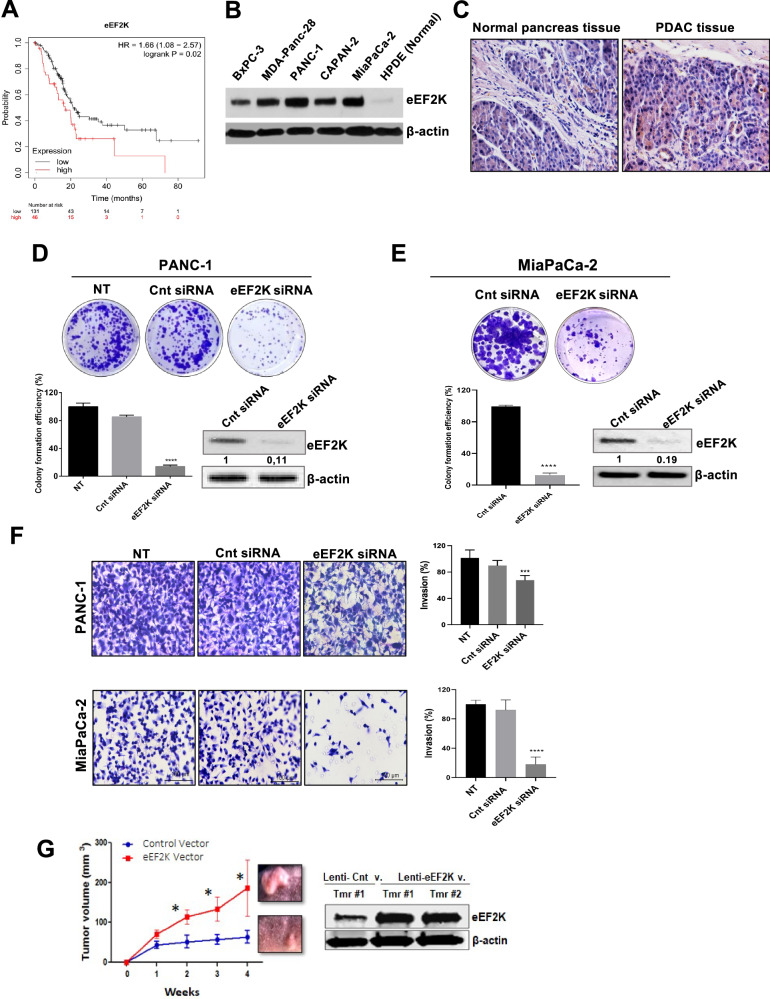
eEF2K expression is associated with poor overall survival in PDAC patients and aggressive properties of pancreatic cancer cells. **A** Higher expression of eEF2K is correlated with poor overall survival (OS) in PDAC patients as determined by Kaplan-Meier survival analysis (*P* = 0.02). The numbers of patients with low and high eEF2K expression are presented at the bottom of the graph. Mo, months. **B** eEF2K expression in PDAC cell lines (BxPC-3, MDA-Panc-28, PANC-1, Capan2, MiaPaCa-2) compared to normal pancreatic duct epithelial (HPDE) cells was determined by Western blot analysis. **C** eEF2K expression in pancreatic cancer patient tissues and normal tissues was determined by immunohistochemistry. **D, E** eEF2K knockdown by eEF2K siRNA in PANC-1 and MiaPaCa-2 suppressed the colony-forming capability of the PDAC cells. Upper panels show representative images of the colonies and lower panels show quantification of the number of colonies formed (****P < 0.0001, relative to cnt siRNA). The data in the bar graphs represent the mean of three independent experiments. **F** Knockdown of eEF2K by siRNA inhibits in vitro invasion capability of PDAC cells in Matrigel (***P < 0.001). Bar graphs represent the mean of three independent experiments and seven fields per sample. **G** Lenti-based ectopic overexpression of human eEF2K gene in PANC-1 cells promoted in vivo tumor xenografts (*n* = 5 mice/group, *P < 0.05). eEF2K overexpression was confirmed in PDAC tumor xenografts obtained from PANC-1 cell-bearing tumors by Western blot. β-actin was used as a loading control. Cnt: control; v.: vector. Error bars represent ± Standard Deviation (SD).

To investigate the clinical significance and prognostic value of eEF2K expression in PDAC patients, we first analyzed The Cancer Genome Atlas (TCGA) database. Kaplan-Meier survival analysis revealed that patients with high eEF2K expression had a shorter overall survival (OS) (*P* = 0.02; Fig. 1A) (low expression *n* = 131, high expression *n* = 46). We further assessed eEF2K protein expression in pancreatic cancer cell lines, normal pancreatic epithelial cells, and patient tissue samples using Western blot and immunohistochemistry. Our results showed high eEF2K expression in pancreatic cancer cell lines, with weak expression in normal human pancreatic ductal epithelial (HPDE) cells (Fig. 1B). Higher eEF2K expression was also observed in tumor tissues of PDAC patients compared to normal tissues (Fig. 1C).

### eEF2K induces cell proliferation, migration, and invasion in PDAC cells

Given the significant upregulation of eEF2K in PDAC cells compared to normal pancreatic cells and tissues, and its association with poor patient survival, we examined its role in cell proliferation, migration, and invasion by knocking down eEF2K expression using specific siRNA in PDAC cells. Knockdown of eEF2K resulted in a substantial decrease in cell proliferation and colony formation in both PANC-1 (****P < 0.0001; Fig. 1D) and MiaPaCa-2 cells (****P < 0.0001; Fig. 1E). Additionally, eEF2K inhibition significantly reduced cell motility and migration in PANC-1 (Fig. S1A) and MiaPaCa-2 cells (*P < 0.05; ****P < 0.0001; Fig. S1B). Similarly, knockdown of eEF2K significantly reduced the invasion capability of PANC-1 (***P < 0.001) and MiaPaCa-2 cells (****P < 0.0001; Fig. 1F).

### eEF2K expression promotes in vivo PDAC tumor growth and its inhibition sensitizes PDAC cells to chemotherapy

To determine the in vivo impact of eEF2K expression in PDAC, we overexpressed eEF2K in PANC-1 cells using a lentiviral vector system. PANC-1 cells transduced with either lenti-control vector or lenti-eEF2K vectors were injected into the flanks of nude mice and monitored weekly. Overexpression of eEF2K resulted in significantly larger tumors in mice (*P < 0.05; Fig. 1G). Western blot analysis confirmed eEF2K overexpression in tumor xenografts from these mice (Fig. 1G). Consistently, eEF2K overexpression enhanced cell migration (Fig. S1C).

We also evaluated whether eEF2K expression influences chemotherapy response. PDAC cells were treated with control or eEF2K siRNA for 24 hours, followed by gemcitabine (0.2–12.5 µM) for an additional 72 hours. eEF2K inhibition significantly increased the efficacy of gemcitabine in PDAC cells (Fig. S2A and S2B).

### In vivo inhibition of eEF2K suppresses the growth of PDAC tumor xenografts in mice

**Fig. 2:**
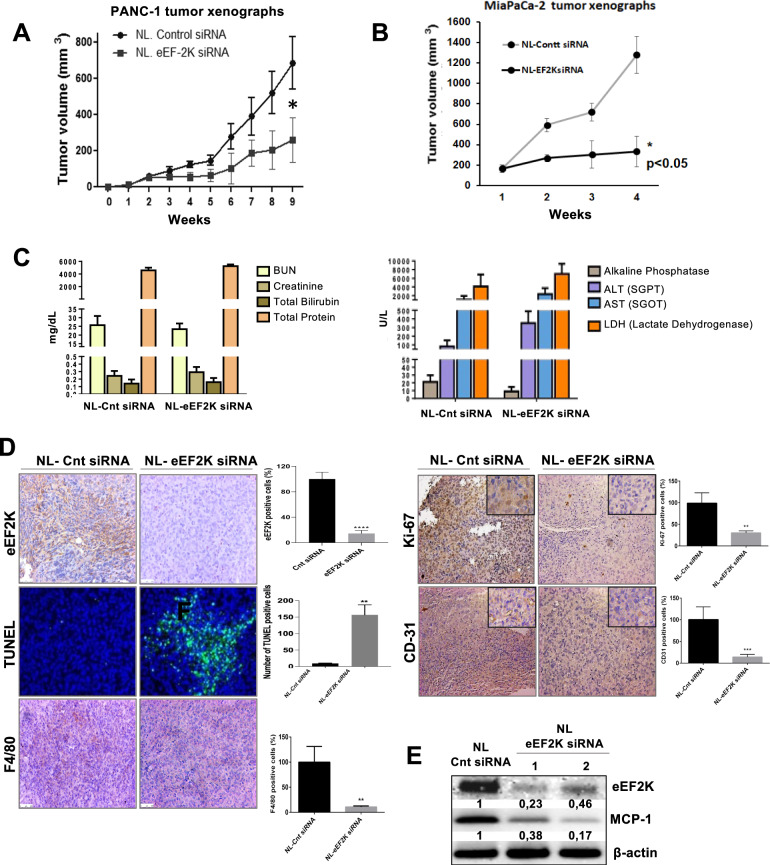
In vivo targeting of eEF2K suppresses tumor growth and decreases MCP-1 expression in orthotopic mouse models of PDAC. **A, B** PANC-1 or MiaPaCa-2 tumor-bearing mice were injected (intravenously from the tail vein) with nanoliposomes (NL) incorporating eEF2K siRNA (0.3 mg/kg, *n* = 5 mice) or control siRNA (0.3 mg/kg, *n* = 5 mice), once per week, and tumor size was measured weekly by a caliper and mean values presented for each group (*P < 0.05). **C** The possible toxic effects of eEF2K silencing were investigated by measuring some parameters related to kidney and liver functions (BUN, creatinine, total bilirubin, total protein, alkaline phosphatase, ALT, AST, LDH levels). **D** Tumor samples from the NL-control siRNA and NL-eEF2K siRNA-treated mice were stained with eEF2K and specific antibodies to detect Ki67, an intratumoral proliferation marker, and CD31, an endothelial marker for evaluation of angiogenesis, and F4/80 for the infiltration of pro-tumorigenic M2 macrophages in PANC-1 tumors. TUNEL staining used for detection of apoptotic cells. Eight randomly selected fields were counted and analyzed for each treatment group (*n* = 5 mice per group). (Magnification, 20x). TUNEL-positive cells stained (green) in both treatment groups were counted and quantified. Nuclei were stained with DAPI (blue) (**P < 0.01, ***P < 0.001). **E** Western blot analysis of MCP-1 expression in tumor samples after in vivo targeting of eEF2K. Error bars represent ± SD.

To evaluate the therapeutic potential of eEF2K inhibition in PDAC and its role in tumorigenesis, we administered nanoliposomal (NL) particles carrying eEF2K siRNA or control siRNA to athymic nude mice bearing PANC-1 or MiaPaCa-2 tumor xenografts. Approximately two weeks after implantation of PDAC cells, mice were treated via tail vein injection with NL particles carrying eEF2K siRNA (0.3 mg/kg, *n* = 5) or control siRNA (0.3 mg/kg, *n* = 5), as detailed in the Methods section. Tumor volumes of PANC-1 and MiaPaCa-tumor xenografts after treated with NL-eEF2K siRNA were significantly smaller compared to those in the NL-control siRNA group (*P < 0.05; Fig. 2A, B, respectively). Furthermore, in vivo NL-eEF2K siRNA treatment was well tolerated and showed no signs of toxicity, as confirmed by blood chemistry toxicity markers (Fig. 2C).

Tumor samples collected from NL-control or NL-eEF2K siRNA-treated mice were stained with specific antibodies against Ki-67, an intratumoral cell proliferation marker, and CD31 for angiogenesis, as well as subjected to TUNEL staining for the detection of apoptotic cells. The results showed that the number of Ki-67 and CD31 positive cells dramatically decreased in the tumor tissues after NL-eEF2K siRNA treatments of mice (**P < 0.01, ***P < 0.001; Fig. 2D). Also, the number of TUNEL-positive apoptotic cells markedly increased after NL-eEF2K siRNA treatments (**P < 0.01; Fig. 2D**)**.

### In vivo targeting of eEF2K reduces MCP-1 expression and M2-TAMs accumulation in PDAC tumors

We also evaluated whether inhibiting eEF2K in vivo decreases the recruitment and density of M2-type pro-tumorigenic TAMs in tumors. Notably, tumors treated with NL-eEF2K siRNA had significantly reduced eEF2K and MCP-1 expression, a marker of monocyte recruitment and macrophage differentiation (Fig. 2E). eEF2K silencing also lowered M2-TAMs infiltration, as indicated by the M2-TAM (mouse) marker F4/80 (**P < 0.01), suggesting that eEF2K regulates M2-TAM accumulation in PDAC tumors (Fig. 2D).

### TAMs are associated with poor overall survival in PDAC patients

To assess the clinical significance of TAMs, we analyzed TCGA data for M2 macrophage-specific markers (CD204, CD163, and CD206). Kaplan-Meier survival analysis indicated that high expression levels of CD206, CD163, and CD204 (Fig. S3A-C) are associated with poor prognosis. Furthermore, co-expression of eEF2K and macrophage markers is significantly associated with shorter OS in PDAC patients (*n* = 54 vs. *n* = 25, respectively) (*P* = 0.00004; Fig. S3D).

### TAMs induce cell migration and invasion through eEF2K in PDAC cells

**Fig. 3:**
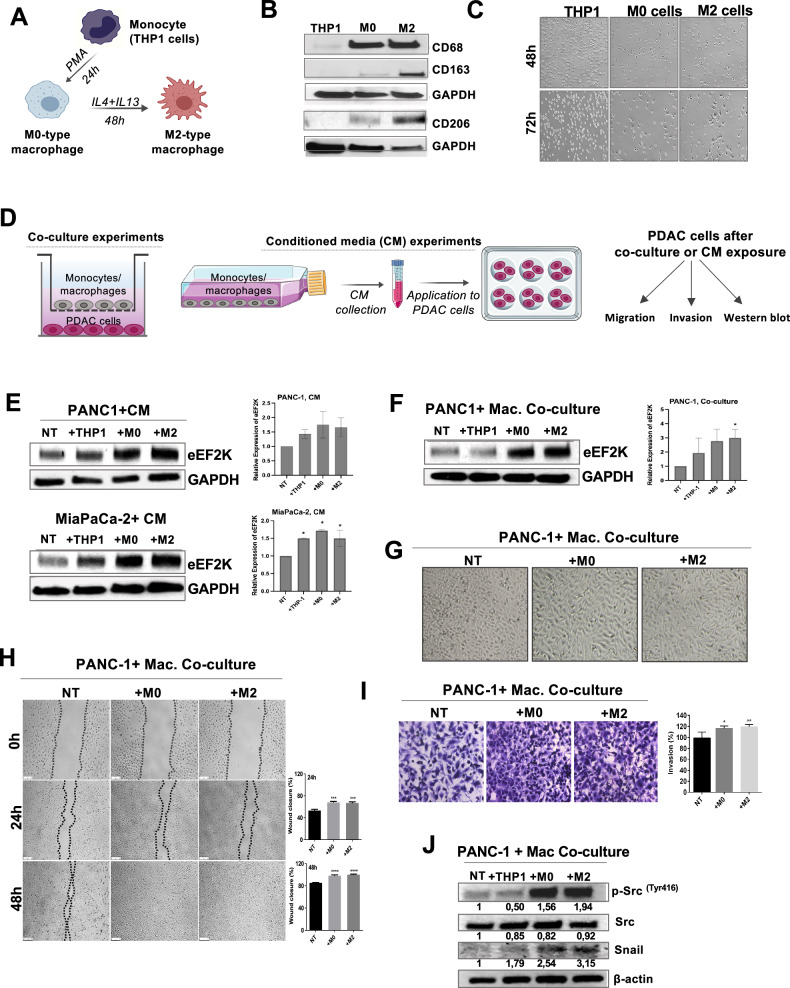
Co-culture of PDAC cells with macrophages induces eEF2K expression, migration, and invasion capabilities of cancer cells. **A–C** Schematic illustration of monocyte-macrophage differentiation and characterization of macrophage differentiation. **B** Monocyte-macrophage differentiation was confirmed by Western blot. CD68 was used as a pan-macrophage marker. CD163 and CD206 were used for the confirmation of M2-type macrophage differentiation. **C** Morphological changes during monocyte-macrophage differentiation were observed under phase-contrast microscope. While monocytes grew in suspension, they became adherent when they polarized into macrophages. M2 pro-tumorigenic macrophages were observed to have a more fibroblast-like phenotype compared to M0 mature macrophages. **D** Illustration of co-culture and conditioned media experiments to investigate the interactions between PDAC cells and monocytes/macrophages. **E** The effect of monocyte/macrophage-derived conditioned media (CM) on eEF2K expression of pancreatic cancer cells (PANC-1 and MiaPaCa-2) was evaluated by Western blot (*n* = 2, *P < 0.05). **F** The impact of co-culture conditions on eEF2K expression of PANC-1 cells. The changes in eEF2K expression in PANC-1 cells were measured by Western blot after 48 hours of co-culture with monocytes/macrophages (*n* = 2, *P < 0.05). **G** The changes in cell morphology after co-culture of PANC-1 cells with macrophages were evaluated through phase-contrast microscopy. **H** The effect of macrophages on the migration capability of PANC-1 cells was evaluated by wound healing assay (***P < 0.001, ****P < 0.0001, relative to NT). **I** Cell invasion was evaluated by Matrigel invasion assay in PANC-1 cells (*P < 0.05, **P < 0.01, relative to NT). **J** Co-culture of macrophages with PANC-1 cells induce signaling pathways and mediators of cell proliferation, survival, cell motility/invasion and epithelial-mesenchymal transition (EMT), including p-Src and Snail detected by Western blot analysis. β-actin and GAPDH were used as loading controls. NT: non-treated; THP-1: monocytes; M0: mature macrophages; M2: tumor-promoting macrophages; CM: conditioned media. Error bars represent ± SD.

Given the poorer prognosis associated with macrophage infiltration and eEF2K expression, we investigated the paracrine effects of macrophages on eEF2K expression and PDAC cell behavior. For this purpose, we induced differentiation of THP-1 cells into M0 and M2 macrophages and confirmed their polarization by assessing the expression of markers CD68, CD163, and CD206, as well as observing morphological changes (Fig. 3A-C).

We then evaluated the impact of macrophage-conditioned media (CM) and the co-culture of macrophages with PDAC cells on eEF2K expression by performing functional assays, as illustrated in Fig. 3D. CM from M0 and M2 macrophages significantly induced eEF2K expression in PANC-1 and MiaPaCa-2 cells (Fig. 3E), but not in PSCs (Fig. S4A).

Indirect co-culture experiments showed that macrophages increased eEF2K expression in PANC-1 cells (Fig. 3F) and PSCs (Fig. S4A) suggesting that TAM-released factors upregulate eEF2K. The differential response of PANC-1 and MiaPaCa-2 cells to macrophage interaction may be related to their molecular differences. A study comparing PANC-1 and MiaPaCa-2 cells indicates that they have different protein expression or mutation patterns. For instance, MiaPaCa-2 cells express E-cadherin, while PANC-1 cells do not, making these cells more metastatic [41]. Another study has shown that PANC-1 and MiaPaCa-2 cells have differentially expressed miRNAs [42], which may also affect their interaction with other cell types, such as macrophages.

Next, we evaluated the effects of co-culture with macrophages on migration, invasion, and related oncogenic pathways in PDAC cells. Consistent with eEF2K induction, macrophages induced mesenchymal-like morphological changes in pancreatic cancer cells, suggesting the induction of epithelial-mesenchymal transition (EMT) due to bidirectional interactions between these cells (Fig. 3G). In addition, co-culture with macrophages significantly increased PANC-1 cell migration (***P < 0.001, ****P < 0.0001) and invasion in Matrigel in Boyden chambers (*P < 0.05, **P < 0.01) after 24 hours and 48 hours of co-culture (Fig. 3H and I). Consistently, the expression of proteins promoting cell migration, invasion, and EMT, such as p-Src and Snail, was induced when PANC-1 cells were co-cultured with macrophages (Fig. 3J).

### TAM-derived exosomes induce eEF2K expression, cell migration, and invasion in PDAC cells

**Fig. 4:**
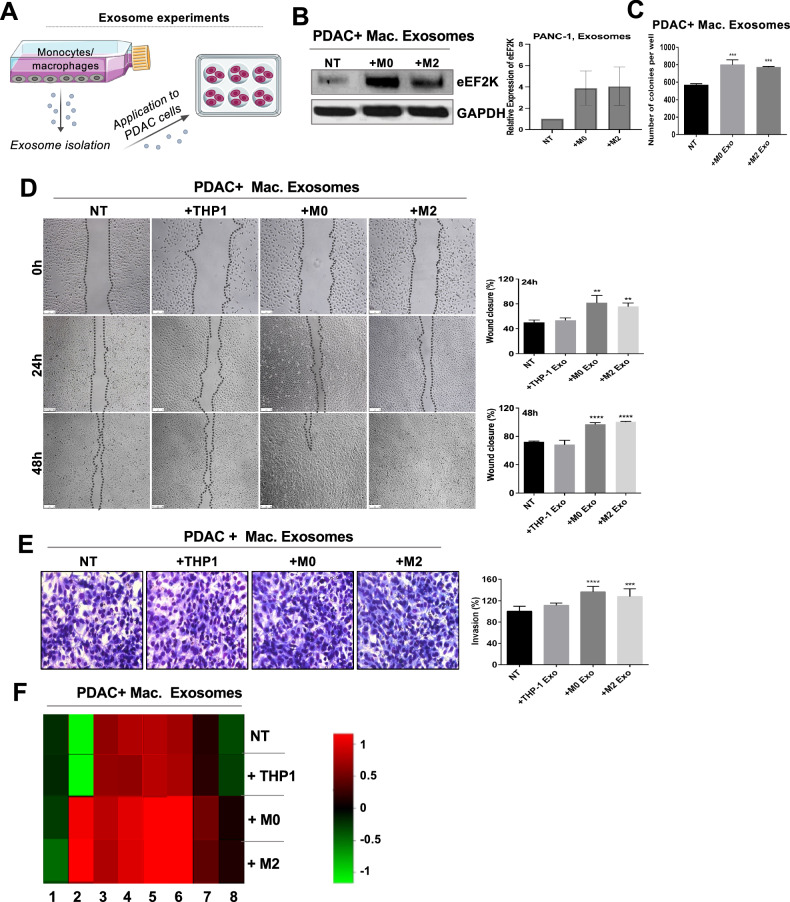
Macrophage-derived exosomes induce eEF2K expression, cell migration, and invasion in PDAC cells. **A** Schematic illustration of exosome experiment design. **B** Macrophage-derived exosomes induced eEF2K expression, **C** cell proliferation and colony formation, **D** cell migration and **E** invasion of PANC-1 cells compared to non-treated cells (NT) (*P < 0.05, **P < 0.01, ***P < 0.001, ****P < 0.0001, respectively). All experiments were independently confirmed at least two times. **F** Heat map of unbiased RPPA analysis of PANC-1 cells after exposure to the monocyte or macrophage-derived exosomes. Average log value from fold-change in the expression of indicated proteins identified from a pool of 305 primary antibodies such as 1. MAPK (p-ERK-T202-Y204); 2. Vimentin; 3. YAP (pS127); 4. AMPK (pT172); 5. eIF4G; 6. Myosin-IIa (pS1943); 7. Stat3 (pY705) and 8. B-Raf (pS445). NT: non-treated; THP-1: monocytes; M0: mature macrophages; M2: tumor-promoting macrophages. Error bars represent ± SD.

Exosomes, nano-vesicles with a diameter of 30 to 120 nm, play a significant role in intercellular communication within the tumor microenvironment and can promote tumor growth and progression through induction of cell proliferation, invasion, and metastasis and reprogramming of the TME [43, 44]. We investigated the role of macrophage-derived exosomes in eEF2K induction and PDAC cell aggressiveness. Briefly, exosomes were isolated from the culture media of THP-1 cells, M0 and M2 macrophages. Then, PANC-1, MiaPaCa-2, and PSC cells were treated with exosomes at a concentration of 1.5 × 10^8^ exosomes/ml for 48 hours, as illustrated in Fig. 4A. The characterization, size, and concentration of exosomes were performed by Nanosight NS300 (Fig. S5A-C).

We showed that eEF2K expression levels increased in PANC-1 cells (Fig. 4B), PSCs (Fig. S4A), and MiaPaCa-2 cells (Fig. S4B) in the presence of macrophage-derived exosomes. Macrophage-derived exosomes also enhanced colony formation (***P < 0.001, Fig. 4C), migration (**P < 0.01, Fig. 4D), and invasion (***P < 0.001, ****P < 0.0001, Fig. 4E) in PANC-1 cells. Similarly, macrophages induced the invasion capability of PSCs (Fig. S4C). RPPA analysis further indicated that macrophage-derived exosomes induced changes in the expression levels of several proteins associated with aggressive characteristics in cancer cells, including MAPK (ERK), vimentin, AMPKa, eIF4G, STAT3, and B-Raf in PANC-1 cells (Fig. 4F**)**.

### Knockdown of eEF2K leads to reduced MCP-1 and Gas6 expression in PDAC cells

**Fig. 5:**
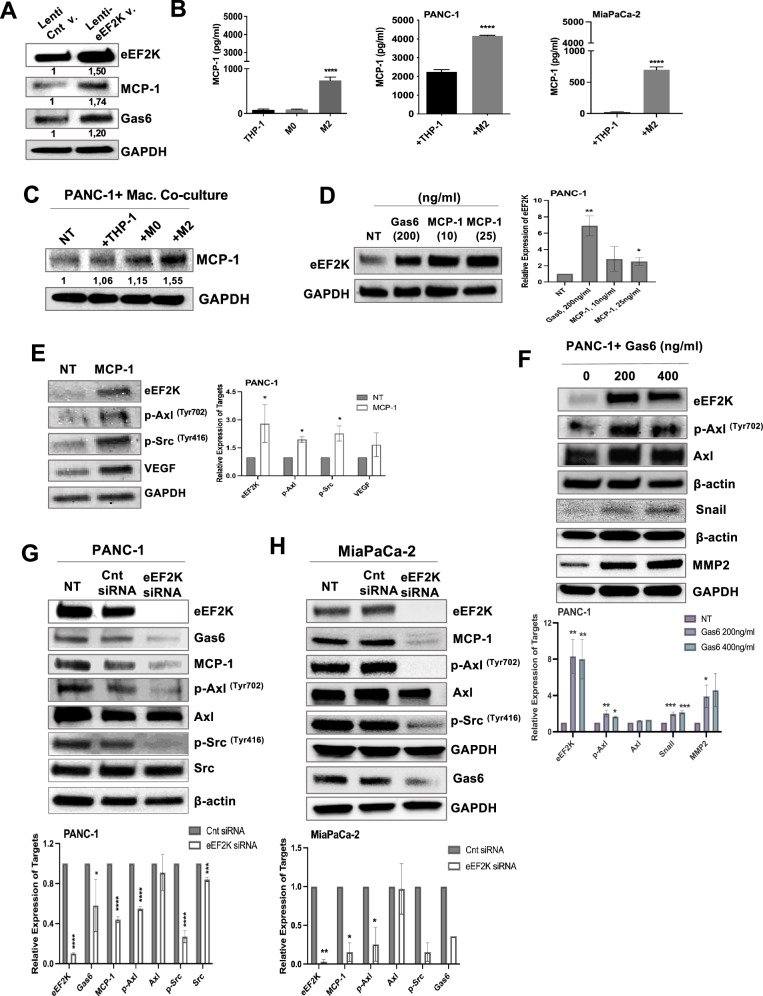
Macrophage-derived factors MCP-1 and Gas6 increase in eEF2K overexpressed cells and these factors, in turn, induce eEF2K expression in PDAC cells. **A** Increased MCP-1 and Gas6 protein expressions were detected in eEF2K-overexpressed cells. **B** Released MCP-1 protein levels in THP-1, M0-type, and M2-type macrophages were measured by ELISA (****P < 0.0001). Besides, MCP-1 levels were measured after co-culturing of PANC-1 or MiaPaCa-2 cells or PSCs with monocytes/macrophages after 48 hours. (****P < 0.0001). **C** Intracellular MCP-1 levels were measured via Western blot in PANC-1 cells following 48 h co-culture with monocytes or macrophages. **D** eEF2K protein expression levels were measured by Western blot in PANC-1 cells after Gas6 and MCP-1 exposure (30 minutes and 24 hours, respectively) (*n* = 2, *P < 0.05, **P < 0.01). **E** The changes in the expression of eEF2K, p-Axl^Tyr702^, p-Src^Tyr416^, and VEGF in PANC-1 cells after 24 hours of MCP-1 treatment (*n* = 2, *P < 0.05). **F** The effects of different concentrations of Gas6 on the expression of eEF2K, p-Axl^Tyr702^, total Axl, Snail, MMP-2, p-eEF2^Thr56^ protein levels were evaluated by Western blot (*n* = 2, *P < 0.05, **P < 0.01, ***P < 0.001). **G**, **H** The changes in the expression of eEF2K, Gas6, MCP-1, p-Axl^Tyr702^, total Axl, p-Src^Tyr416^, total Src, and integrin β1 in PDAC cells following transfection with eEF2K siRNA for 48 h (*n* = 2, *P < 0.05, **P < 0.01, ***P < 0.001, ****P < 0.0001). Error bars represent ± SD.

Since we found that increased eEF2K expression drives cell proliferation, invasion, and tumor growth, we next examined the interplay between eEF2K and TAM-derived factors such as MCP-1, a key chemokine involved in the recruitment and tumoral infiltration of monocytes [24] as well as monocyte-macrophage differentiation [25, 26]. Similarly, we focused on another critical factor, Gas6, which is released by various cell types in the TME, including macrophages. Cancer cells are known to stimulate Gas6 production in macrophages to fuel cancer cell proliferation [45]. Increased Gas6 expression further induces cancer aggressiveness and release of factors that can trigger more Gas6 production, creating a kind of vicious cycle. Therefore, we explored the interactions between eEF2K and TAM-derived factors, MCP-1 and Gas6. Overexpression of eEF2K in PANC-1 cells led to increased MCP-1 and Gas6 expression (Fig. 5A). MCP-1, primarily released by M2 macrophages, was significantly increased under co-culture conditions (****P < 0.0001; Fig. 5B). Intracellular MCP-1 levels were also found to be elevated in PANC-1 cells in the presence of macrophages (Fig. 5C).

### MCP-1 and Gas6 induce eEF2K expression in PDAC cells

To confirm the direct effect of MCP-1 and Gas6 on eEF2K expression, we treated PDAC cells with Gas6 and MCP-1 for 30 minutes and 24 hours, respectively, and eEF2K expression was subsequently analyzed. As shown in Fig. 5D, eEF2K expression was significantly upregulated in PANC-1 cells following exposure to either Gas6 or MCP-1. Treatment with MCP-1 and Gas6 not only induced eEF2K expression but also activated Axl and Src, as evidenced by elevated levels of p-Axl (Thr702) and p-Src (Tyr416), along with upregulated VEGF expression, which are key mediators of cell proliferation, migration/invasion, and angiogenesis, respectively (Fig. 5E). In addition, Gas6 induced the expression of MMP-2 and Snail, which are mediators of cell invasion and epithelial-mesenchymal transition (EMT), respectively (Fig. 5F). MCP-1 also slightly enhanced the colony-forming ability of PANC-1 cells (*P < 0.05; Fig. S4D).

To investigate the bidirectional signaling mechanisms mediated by eEF2K, we knocked down eEF2K in PANC-1 and MiaPaca-2 cells. Knockdown of eEF2K led to a reduction in Gas6, p-Axl, and MCP-1 proteins levels in both PANC-1 (Fig. 5G) and MiaPaCa-2 cells (Fig. 5H). Consistently, silencing of Axl, the receptor for Gas6, decreased the colony formation, migration and invasion abilities of MiaPaCa-2 cells (Fig. S6A-C).

Next, we evaluated whether eEF2K can modulate MCP-1 in TAM cells. Our in vitro studies showed that MCP-1 exposure induced the differentiation of M0 macrophages into M2-TAMs, as indicated by increased expression of CD206, a marker for human M2-TAMs (Fig. S7A). Intriguingly, we also found that MCP-1 treatment drastically induced eEF2K expression in macrophages, while there was no eEF2K expression in M0 macrophages or in interleukin-stimulated M2 macrophages (Fig. S7A). Consistently, eEF2K silencing reduced the expression of MCP-1 receptor, CCR2, in M2 macrophages, providing further evidence for the existence of a positive feedback cycle between eEF2K and MCP-1/CCR2 axis (Fig. S7B). Furthermore, eEF2K inhibition in M2-TAMs reduced Gas6 expression (Fig. S7B), indicating potential crosstalk between Gas6 and eEF2K.

**Fig. 6:**
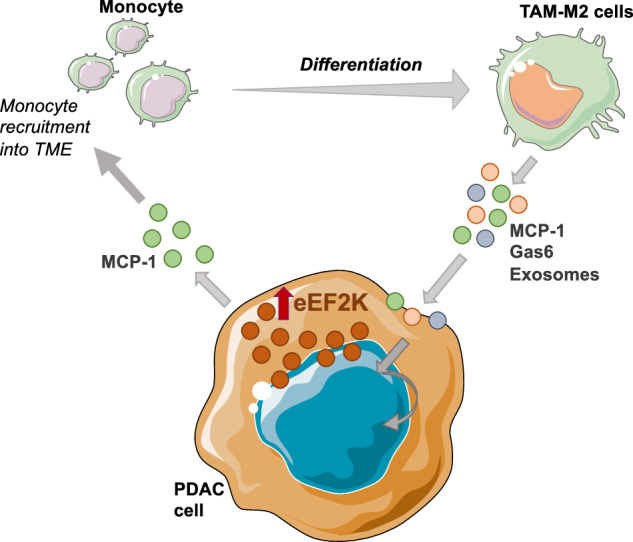
eEF2K-mediated regulation of the TME. Schematic representation of a proposed positive feedback loop between tumor cells and TAMs, mediated by the eEF2K/MCP-1 axis. This loop promotes the recruitment and accumulation of monocytes/TAMs, contributing to an immunosuppressive, pro-tumorigenic TME and enhancing pancreatic tumor progression.

As a result, our findings reveal a novel regulatory feedback loop involving eEF2K, MCP-1, and Gas6 in PDAC tumors, which promotes the accumulation of M2-TAMs and enhances tumor aggressiveness (Fig. 6). Therefore, targeting the eEF2K/MCP-1/Gas6 axis represents a promising therapeutic strategy to disrupt this cycle and suppress PDAC progression.

## Discussion

This study presents novel findings that eEF2K expression is linked to poor prognosis and shorter survival in PDAC patients. eEF2K promotes tumor growth and progression and more importantly, in vivo inhibition of eEF2K led to significant suppression of tumor growth in multiple PDAC tumor models in mice with no observed toxicity, suggesting that eEF2K is an actionable novel target in PDAC. Furthermore, our findings highlight the critical role of eEF2K in mediating complex crosstalk between PDAC cells and macrophages, thereby reprogramming the TME and inducing recruitment and accumulation of TAMs, which also correlates with poor patient survival.

We demonstrate for the first time that bidirectional interaction between pancreatic cancer cells and macrophages leads to upregulation of eEF2K signaling axis, which contributes to PDAC cell proliferation, migration, invasion, and accumulation of M2-type TAMs. Our findings reveal that eEF2K expression in PDAC cells is induced through communication with M2-TAMs via secretion of MCP-1, Gas6, and exosomes. Furthermore, ectopic expression of eEF2K in PDAC cells leads to increased expression of monocyte chemoattractants such as MCP-1 and Gas6, suggesting a novel positive feedback loop between PDAC cells and TAMs. This loop perpetuates M2-TAMs accumulation, further inducing eEF2K and promoting pancreatic cancer growth and progression. Notably, in vivo targeting of the eEF2K/MCP-1 signaling axis with siRNA-nanotherapeutics inhibits tumor growth, induces apoptosis, and reduces the accumulation of pro-tumorigenic M2-TAMs in orthotopic mouse models of PDAC.

eEF2K, an atypical protein alpha kinase [32, 46], is known to slow down protein synthesis by phosphorylating its substrate, eEF2, on Thr56. This phosphorylation reduces the affinity of eEF2 for ribosomes, thereby inhibiting the elongation phase of protein synthesis [32]. This process is critical under stress conditions, allowing cells to adapt to fluctuations in nutrient and energy availability [47]. Beyond its role in protein synthesis regulation, eEF2K activates clinically significant pathways, such as PI3K/AKT, cyclin D1, and c-Myc, which promote cancer cell proliferation, invasion, and tumor growth in various types of cancers, including triple-negative breast (TNBC) [36, 48, 49] lung [50], ovarian [38], and pancreatic cancer [37].

Based on data from the literature, eEF2K promotes aggressive features of cancer cells through several mechanisms. It protects cancer cells from cell death due to energy or nutrient deprivation in the TME by slowing down protein synthesis [51]. Additionally, eEF2K induces autophagy to provide alternative energy sources for maintaining cancer cell proliferation and survival [36, 52]. However, in some instances, eEF2K can also inhibit protein synthesis without inducing autophagy [53]. eEF2K activates multiple signaling pathways, such as cyclin D1, c-Myc, Bcl-xL and integrin beta-1/Src/FAK, PI3K/Akt and p-ERK/MAPK, indicating that its effects extend beyond the regulation of protein translation [36, 38, 50, 54, 55]. Moreover, eEF2K expression is associated with shorter survival in PDAC patients and other cancers, highlighting its significance as a biomarker for poor prognosis [50, 56, 57].

While eEF2K was initially thought to regulate mRNA translation solely by phosphorylating eEF2, later research has revealed more complex mechanisms underlying eEF2K-induced oncogenic signaling. Studies have shown that PP2A, a tumor suppressor that regulates key phosphoproteins involved in apoptosis, proliferation, and DNA damage response, is also a substrate of eEF2K [58]. PP2A is known to dephosphorylate important molecules such as Akt, p53, c-Myc, β-catenin, p-JNK, FAK, p-38 MAPK, and ERK [59–61]. The regulation of PP2A by eEF2K may contribute to its oncogenic role in pancreatic cancer. Additionally, eEF2K enhances the expression of integrins by promoting the association of their mRNAs with polysomes, suggesting further mechanisms by which eEF2K promotes tumor growth [39]. However, further investigations are needed to elucidate the precise role of eEF2K in pancreatic cancer models.

In addition to its involvement in cell migration, invasion, and tumor growth, we have previously demonstrated that eEF2K induces chemoresistance in breast cancer cells[36] and confers resistance to cellular stress [62]. In the current study, we further showed that the inhibition of eEF2K enhances the cytotoxic effects of gemcitabine in pancreatic cancer cells. This finding underscores the importance of eEF2K as a molecular target in pancreatic cancer, as its inhibition may positively influence the efficacy of gemcitabine, the first-line standard chemotherapy regimen.

Development of pharmacological inhibitors for eEF2K has been challenging due to the unsolved 3D crystal structure of the protein. However, recent studies have focused on developing inhibitors or protein degraders to target eEF2K, using computational modeling approaches which provide partial structural insights into eEF2K’s domains and active sites, discussed in recent reviews [33, 63–65]. Despite these advancements, there are still relatively few reports on eEF2K inhibitors, their functions and the exact mechanisms of action of some inhibitors remain unknown. The pharmacological inhibitors are generally not specific or potent enough for clinical translation [33].

Several natural compounds, such as thymoquinone [66] and rottlerin, have been reported to inhibit eEF2K in various studies, including those from our group [37, 67]. However, these inhibitors, such as rottlerin, lack specificity for eEF2K and inhibit other protein kinases, like PKC-delta, at lower concentrations [68]. Similarly, NH125 was initially reported as a potent and selective inhibitor of eEF2K [69], but subsequent studies revealed that it is not specific to eEF2K and instead induces its phosphorylation [70].

Other eEF2K inhibitor candidates include compound-34 (a thieno[2–3-b] pyridine analogue), 21I (β-phenylalanine derivatives) [71, 72], TS-2 and TS-4 (selenazine derivatives) [73] and TX-1918 [74]. However, some of these compounds, such as TX-1918, lack specificity and also inhibit other tyrosine kinases, including PKA, PKC, and Src-K, ultimately failing to demonstrate anti-cancer activity against several cancer cell lines [74]. Similarly, A-484954 is not a potent eEF2K inhibitor, requiring high concentrations (around 50 µM) for effective inhibition. Emerging approaches, such as proteolysis-targeting chimeras (PROTAC), which promote targeted protein degradation via the proteasome, have been reported. Among these designed molecules, compound 11 l effectively degraded eEF2K and induced apoptosis in breast cancer cell lines [75], though its specificity and mechanism require further investigation. Overall, these findings highlight the need for safer, more potent, and highly specific eEF2K inhibitors for clinical applications.

A growing body of evidence, including our current study, indicates that the presence of M2 phenotype TAMs in tumors is significantly associated with shorter patient survival and poor prognosis. A recent comprehensive meta-analysis further underscored this association, revealing that pancreatic cancer patients with high numbers of CD68^+^TAMs had worse overall survival [76]. In our present study, Kaplan-Meier survival analysis showed that pancreatic cancer patients with elevated levels of TAMs (indicated by the expression of CD163, CD204, and CD206) or eEF2K, as well as those expressing both CD68 and eEF2K, have poorer overall survival. Notably, the co-expression of CD68 and eEF2K correlates with the worst prognosis. Collectively, these findings provide compelling evidence of the role of TAMs in promoting the aggressive nature of cancer cells, characterized by increased proliferation, invasion, migration, angiogenesis, and chemoresistance [77–79].

While it is widely recognized that a bidirectional interaction exists between cancer cells and TAMs, contributing to tumor progression [80–82], the molecular mechanisms underlying this intercellular communication remain poorly understood. Our study sheds light on these mechanisms, showing that M2 macrophages promote a tumor-supportive environment by inducing increased levels of eEF2K in pancreatic cancer cells. This induction occurs through the release of factors such as MCP-1 and Gas6 from TAMs when pancreatic cancer cells are exposed to macrophage-derived exosomes, conditioned media, or are indirectly co-cultured with macrophages.

TAMs release various cytokines, growth factors, and chemokines that facilitate tumor progression. Among these, Gas6 and MCP-1 (CCL2) are particularly significant in the TME due to their roles in monocyte infiltration and differentiation into M2-type macrophages [24–26]. MCP-1 is also known to enhance cancer migration, invasion, and metastasis [27, 83–85], and its expression in solid tumors, including pancreatic cancer, serves as a poor prognostic biomarker associated with shorter patient survival. Consequently, MCP-1 is considered an important therapeutic target in pancreatic cancer treatment [28]. The elevated serum levels of MCP-1 in pancreatic cancer patients further underscore its role in disease progression, making it an important therapeutic target [29, 30].

In co-culture experiments, we observed a significant increase in MCP-1 levels in the supernatant when PDAC cells were co-cultured with M2-type macrophages, suggesting that the interactions between TAMs and PDAC cells contribute to oncogenic signaling and poor prognosis. Additionally, eEF2K expression has been linked to the induction of epithelial-mesenchymal transition (EMT), migration, invasion, and chemoresistance in various cancers, including pancreatic [37], lung [50], breast cancer [36], and glioma [86], further supporting its oncogenic role in solid tumors. Our findings demonstrated increased eEF2K expression in PANC-1 cells co-cultured with TAMs, alongside enhanced colony formation, migration, and invasion. These findings suggest that eEF2K-mediated communication between macrophages and cancer cells fuels cancer aggressiveness.

Our study revealed increased levels of MCP-1 and Gas6 in co-culture conditions, with eEF2K-overexpressing cells exhibiting even higher levels of these factors. This suggests a potential regulatory role for eEF2K in modulating MCP-1 and Gas6 expression. Moreover, treating PANC-1 cells with MCP-1 or Gas6 induced eEF2K expression, highlighting a reciprocal regulatory loop between these molecules. Silencing eEF2K resulted in reduced expression of both MCP-1 and Gas6, further supporting the existence of a feedback loop between eEF2K and these signaling molecules.

We also explored the impact of exosomal communication between cancer cells and macrophages on eEF2K expression and the aggressiveness of cancer cells. Macrophage-derived exosome-mediated communication within the TME has been widely studied to elucidate the impact of exosomes on tumor progression [87, 88]. Here, we showed that macrophage-derived exosomes increased eEF2K expression and enhanced colony formation, migration, and invasion in PANC-1 cells, although no significant effects were observed in MiaPaCa-2 cells. We then focused on PANC-1 cells for the exosome experiments because macrophage-derived exosomes did not induce eEF2K expression as strongly in MiaPaCa-2 cells. Heat map analysis of PANC-1 cells treated with monocyte and macrophage-derived exosomes revealed increased expressions of several proteins. Specifically, we observed elevated levels of MAPK (p-ERK-T202-Y204), Vimentin, YAP (pS127), AMPK (pT172), eIF4G, Myosin-IIa (pS1943), Stat3 (pY705) and B-Raf (pS445), suggesting that M2 macrophage-derived exosomes induce various oncogenic signaling pathways in pancreatic cancer cells.

In summary, while the tumor-promoting role of eEF2K has been reported in several solid cancers, this is the first study demonstrating its multifaceted oncogenic role in promoting tumor growth and progression. Furthermore, our study highlights its contribution to reprogramming the TME by regulating the accumulation of TAMs and the TME-mediated regulation of eEF2K, suggesting a positive feedback loop. Future studies may address the secretome profile of PDAC cells and TAMs, which could facilitate crosstalk between the TME and cancer cells and elucidate the network of signaling pathways involved. Additionally, having identified a novel eEF2K-Gas6-MCP-1 positive feedback loop that enhances eEF2K activity and promotes tumor growth and poor prognosis, we hypothesize that similar signaling loops may exist in other solid cancers.

Overall, our results demonstrate that eEF2K mediates interactions between pancreatic cancer cells and TAMs through MCP-1/Gas6 signaling and exosomal communication, promoting tumor growth and progression. We propose that eEF2K creates a positive feedback loop that facilitates the accumulation of TAMs. Notably, in vivo silencing of eEF2K in PDAC mouse tumor xenografts inhibited tumor growth, decreased MCP-1 expression, and reduced TAM accumulation. Therefore, eEF2K emerges as a promising therapeutic target for pancreatic cancer, with the potential to modulate interactions within the TME interactions and improve patient outcomes.

## Supplementary information

Supplementary data

antibody list

Reproducibility Checklist

## Data Availability

The authors confirm that the data supporting the findings of this study are available within the article and its supplementary material. Raw data that support the findings of this study are available from the corresponding author upon reasonable request. This article is extracted from the doctoral dissertation of Didem Karakas, entitled “Targeting Pancreatic Cancer and Tumor Microenvironment Through Eukaryotic Elongation Factor 2 Kinase (eEF2K) Inhibition”, supervised by Egemen Dere&Bulent Ozpolat (Ph.D. Dissertation, Uludag University, Bursa, Turkiye, 2017, Council of Higher Education Thesis Number: 470558).
