# Supplementary material for: eEF2K is a poor prognostic factor and novel molecular target in pancreatic cancer: regulating tumor growth and progression via the tumor microenvironment
Source: Cell Death Dis. 2025 Jul 7;16(1):501. doi: 10.1038/s41419-025-07803-w (PMC12234705; doi:10.1038/s41419-025-07803-w)
Supplement: Supplementary file 1 — Supplementary data [file 41419_2025_7803_MOESM1_ESM.pptx]

## Slide 1
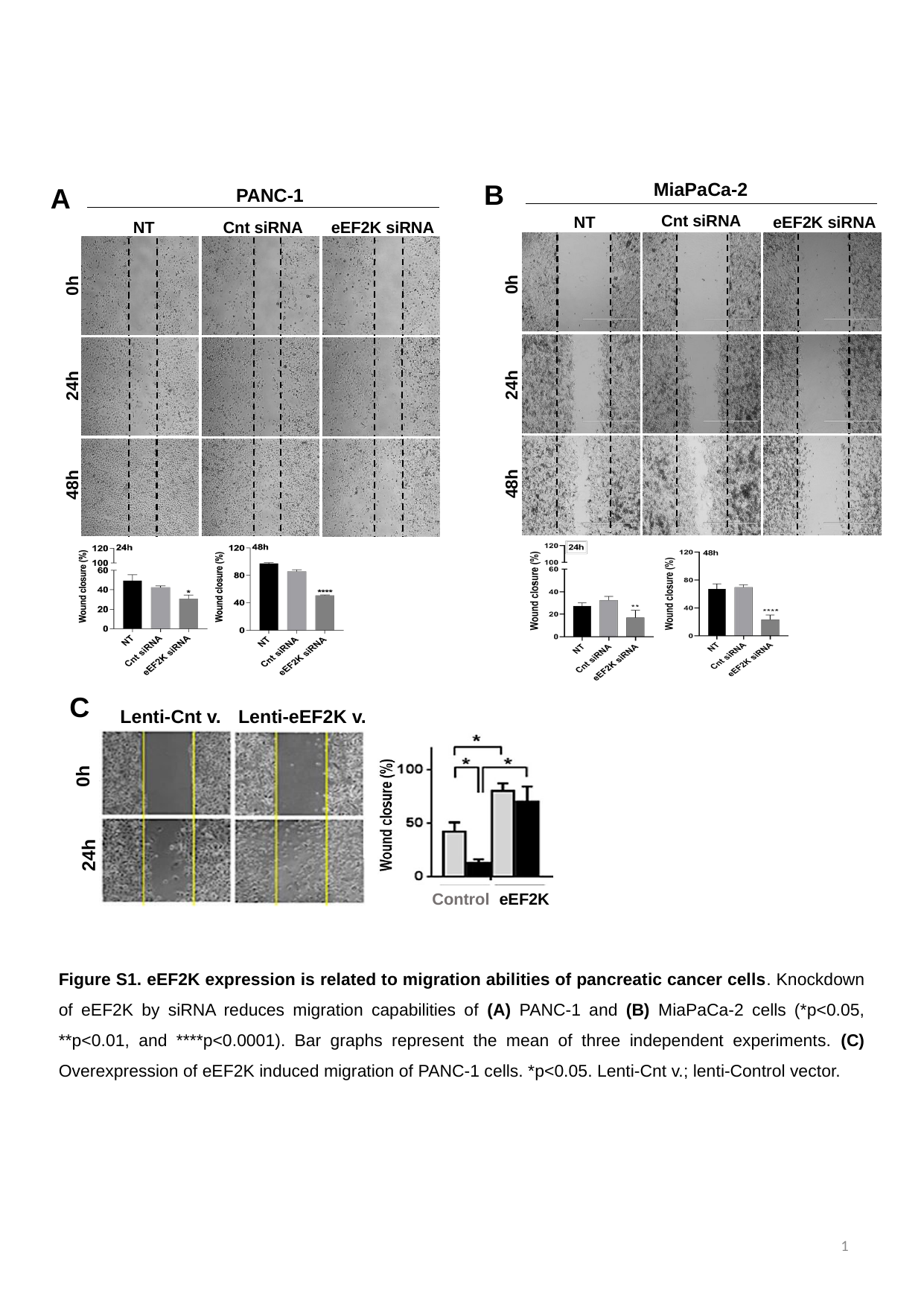

B
MiaPaCa-2
A
PANC-1
NT
Cnt siRNA
eEF2K siRNA
0h
24h
48h
Cnt siRNA
NT
eEF2K siRNA
0h
24h
48h
C
Lenti-Cnt v.
Lenti-eEF2K v.
 0h
24h
eEF2K
Control
Figure S1. eEF2K expression is related to migration abilities of pancreatic cancer cells. Knockdown of eEF2K by siRNA reduces migration capabilities of (A) PANC-1 and (B) MiaPaCa-2 cells (*p<0.05, **p<0.01, and ****p<0.0001). Bar graphs represent the mean of three independent experiments. (C) Overexpression of eEF2K induced migration of PANC-1 cells. *p<0.05. Lenti-Cnt v.; lenti-Control vector.
1

## Slide 2
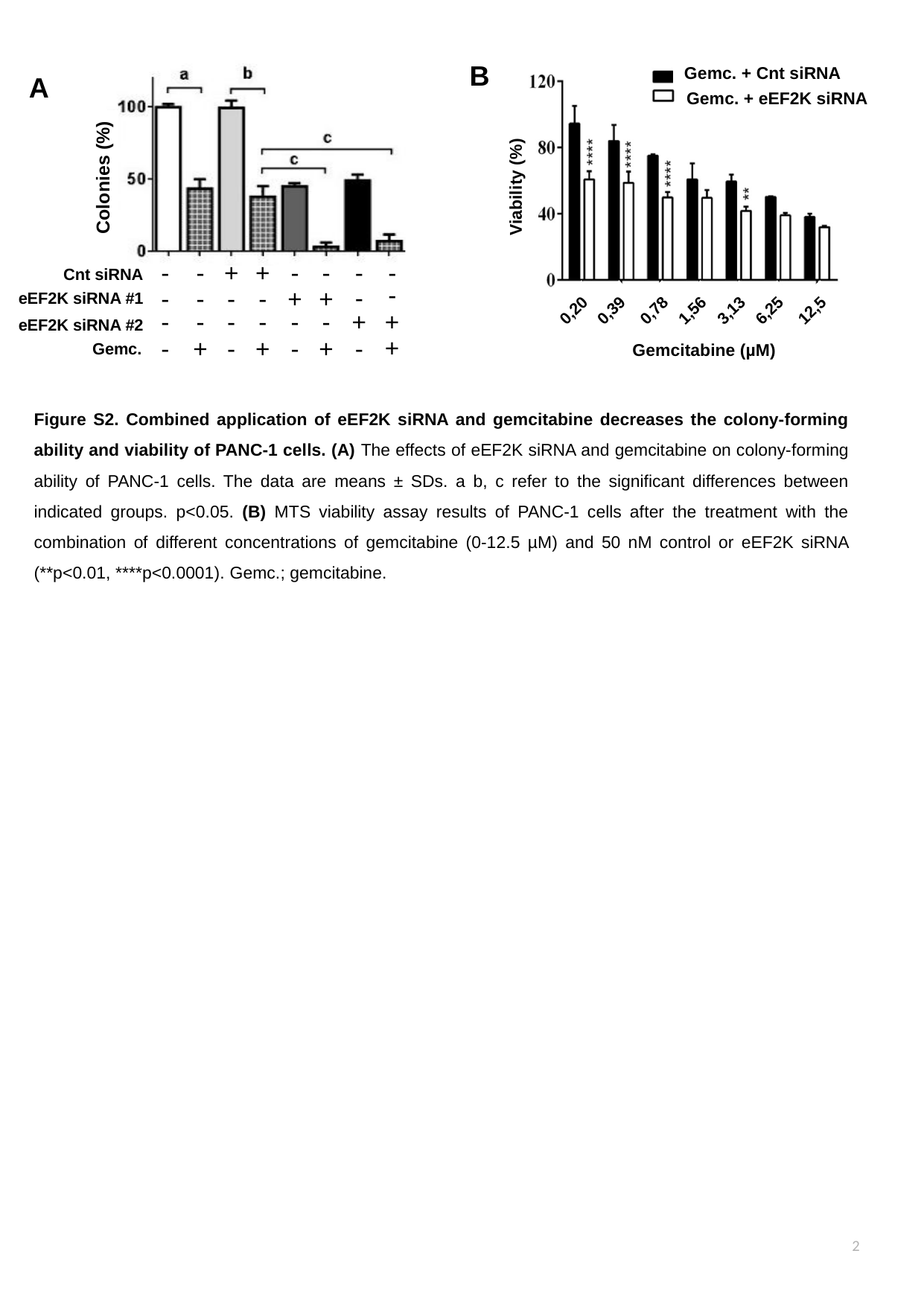

Colonies (%)
-
-
+
+
-
-
-
-
Cnt siRNA
-
-
-
-
-
-
+
+
+
-
-
-
-
-
-
+
+
-
+
-
+
-
+
-
Gemc.
eEF2K siRNA #1
eEF2K siRNA #2
Gemc. + Cnt siRNA
Gemc. + eEF2K siRNA
Viability (%)
Gemcitabine (µM)
B
A
0,20
0,39
0,78
1,56
3,13
6,25
12,5
Figure S2. Combined application of eEF2K siRNA and gemcitabine decreases the colony-forming ability and viability of PANC-1 cells. (A) The effects of eEF2K siRNA and gemcitabine on colony-forming ability of PANC-1 cells. The data are means ± SDs. a b, c refer to the significant differences between indicated groups. p<0.05. (B) MTS viability assay results of PANC-1 cells after the treatment with the combination of different concentrations of gemcitabine (0-12.5 µM) and 50 nM control or eEF2K siRNA (**p<0.01, ****p<0.0001). Gemc.; gemcitabine.
2

## Slide 3
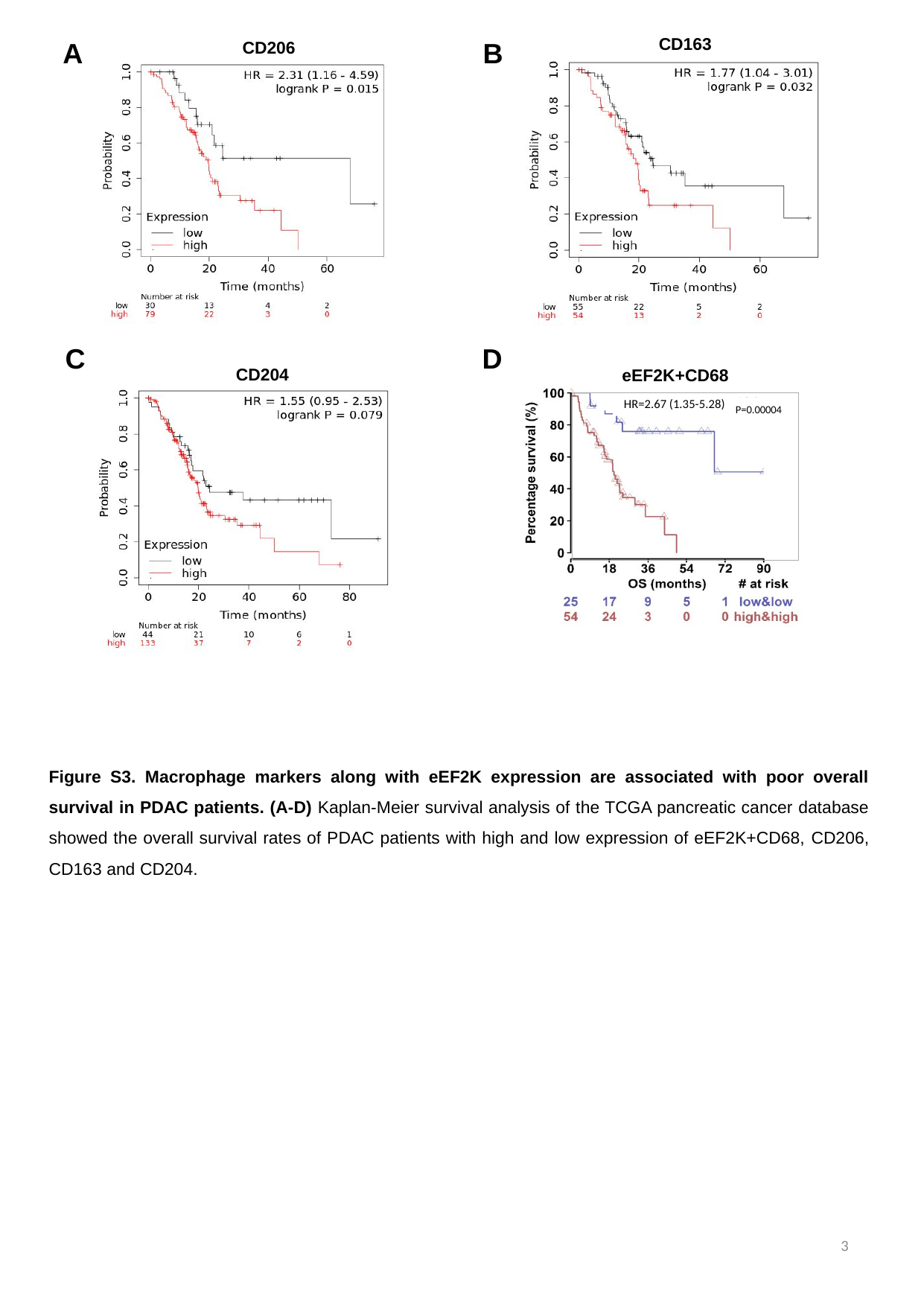

CD163
CD206
A
B
C
D
CD204
 eEF2K+CD68
HR=2.67 (1.35-5.28)
P=0.00004
Figure S3. Macrophage markers along with eEF2K expression are associated with poor overall survival in PDAC patients. (A-D) Kaplan-Meier survival analysis of the TCGA pancreatic cancer database showed the overall survival rates of PDAC patients with high and low expression of eEF2K+CD68, CD206, CD163 and CD204.
3

## Slide 4
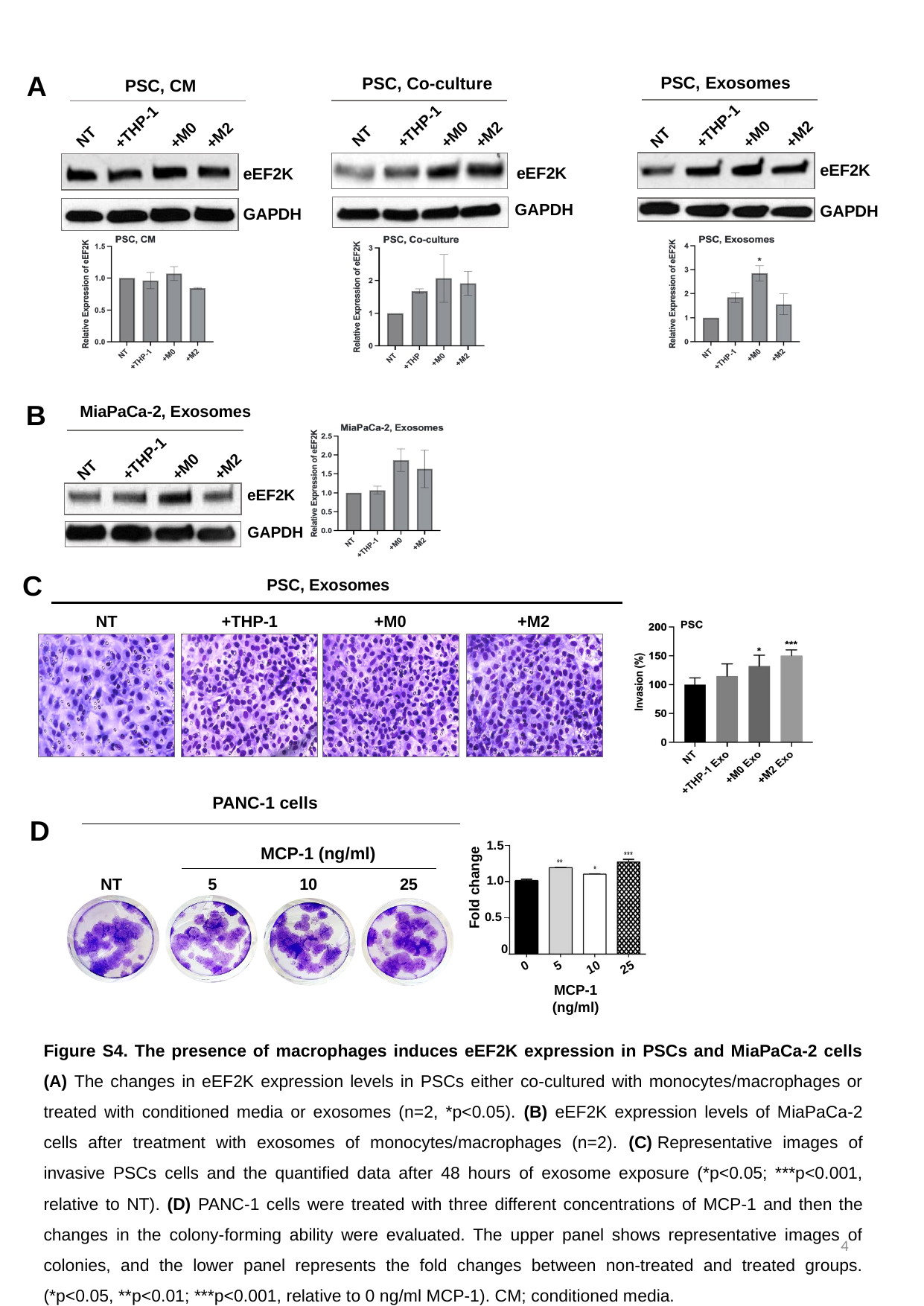

A
PSC, Exosomes
PSC, Co-culture
+THP-1
+M0
+M2
NT
eEF2K
GAPDH
PSC, CM
+THP-1
+M2
+M0
NT
eEF2K
GAPDH
+M0
+THP-1
+M2
NT
eEF2K
GAPDH
B
MiaPaCa-2, Exosomes
+THP-1
+M2
+M0
NT
eEF2K
GAPDH
C
PSC, Exosomes
NT
+THP-1
+M0
+M2
PANC-1 cells
D
Fold change
MCP-1 (ng/ml)
1.0
0.5
0
0
5
10
25
1.5
MCP-1 (ng/ml)
NT
5
10
25
Figure S4. The presence of macrophages induces eEF2K expression in PSCs and MiaPaCa-2 cells (A) The changes in eEF2K expression levels in PSCs either co-cultured with monocytes/macrophages or treated with conditioned media or exosomes (n=2, *p<0.05). (B) eEF2K expression levels of MiaPaCa-2 cells after treatment with exosomes of monocytes/macrophages (n=2). (C) Representative images of invasive PSCs cells and the quantified data after 48 hours of exosome exposure (*p<0.05; ***p<0.001, relative to NT). (D) PANC-1 cells were treated with three different concentrations of MCP-1 and then the changes in the colony-forming ability were evaluated. The upper panel shows representative images of colonies, and the lower panel represents the fold changes between non-treated and treated groups. (*p<0.05, **p<0.01; ***p<0.001, relative to 0 ng/ml MCP-1). CM; conditioned media.
4

## Slide 5
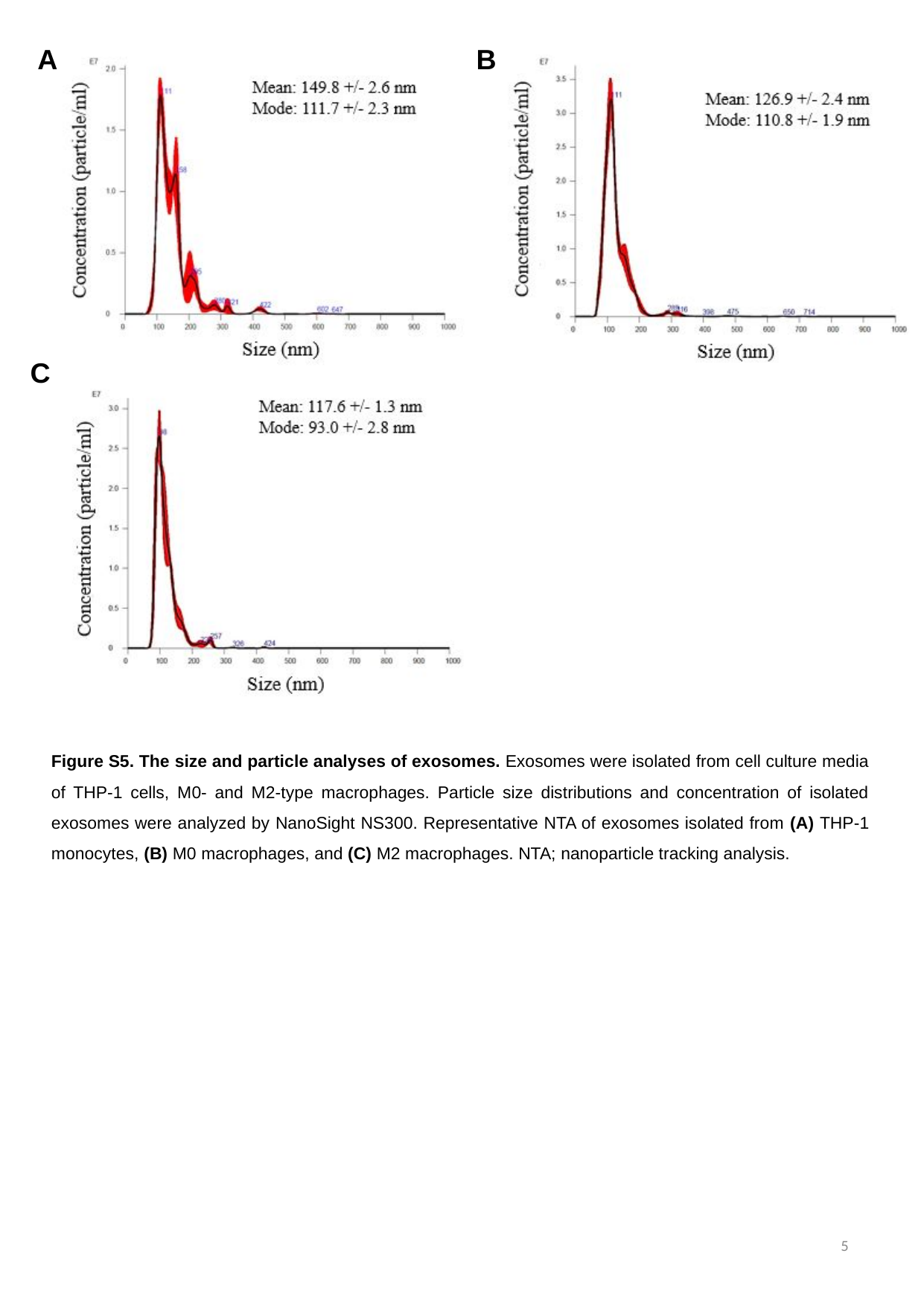

A
B
C
Figure S5. The size and particle analyses of exosomes. Exosomes were isolated from cell culture media of THP-1 cells, M0- and M2-type macrophages. Particle size distributions and concentration of isolated exosomes were analyzed by NanoSight NS300. Representative NTA of exosomes isolated from (A) THP-1 monocytes, (B) M0 macrophages, and (C) M2 macrophages. NTA; nanoparticle tracking analysis.
5

## Slide 6
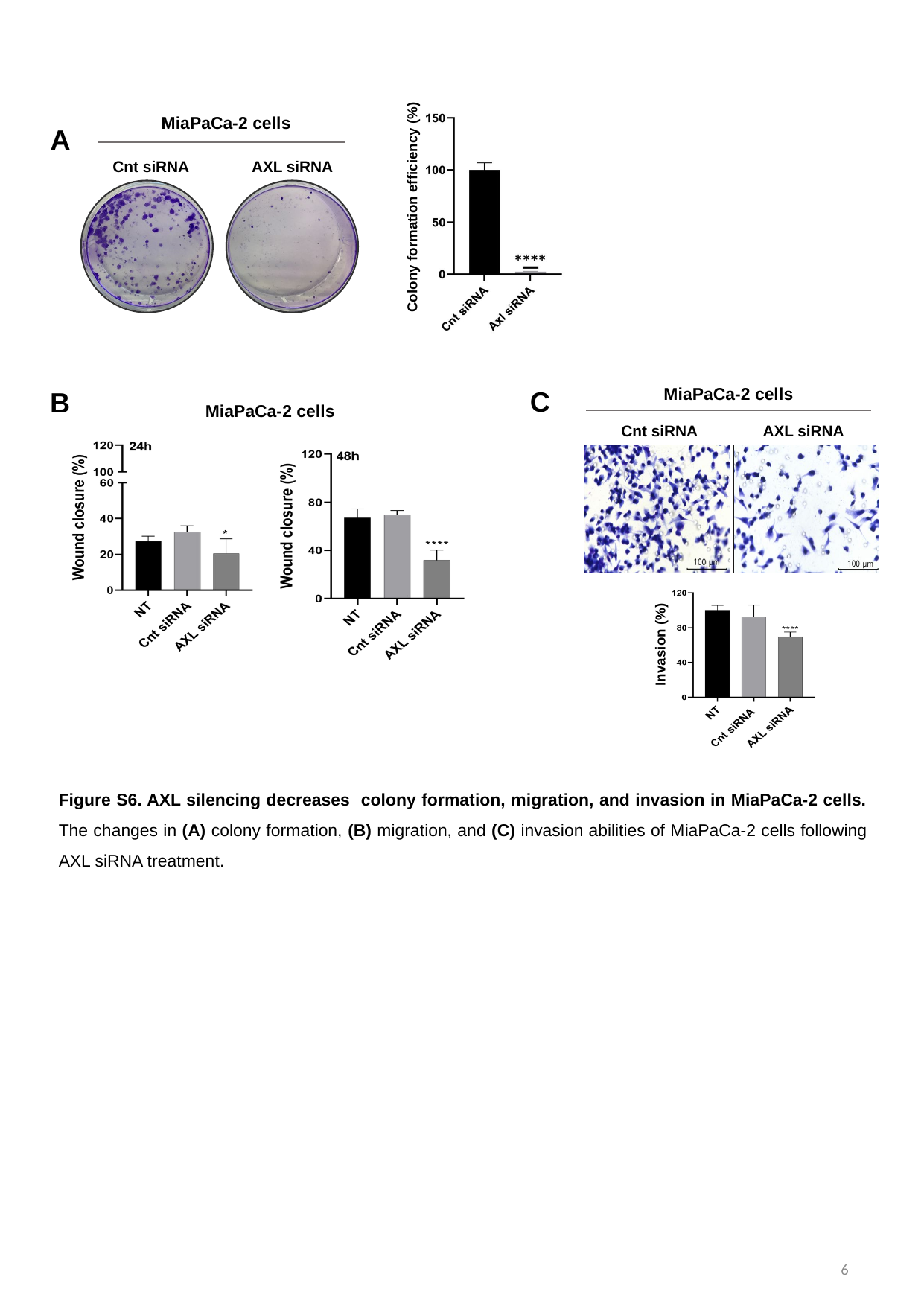

Colony formation efficiency (%)
MiaPaCa-2 cells
A
Cnt siRNA
AXL siRNA
MiaPaCa-2 cells
C
B
MiaPaCa-2 cells
Cnt siRNA
AXL siRNA
Invasion (%)
Figure S6. AXL silencing decreases colony formation, migration, and invasion in MiaPaCa-2 cells. The changes in (A) colony formation, (B) migration, and (C) invasion abilities of MiaPaCa-2 cells following AXL siRNA treatment.
6

## Slide 7
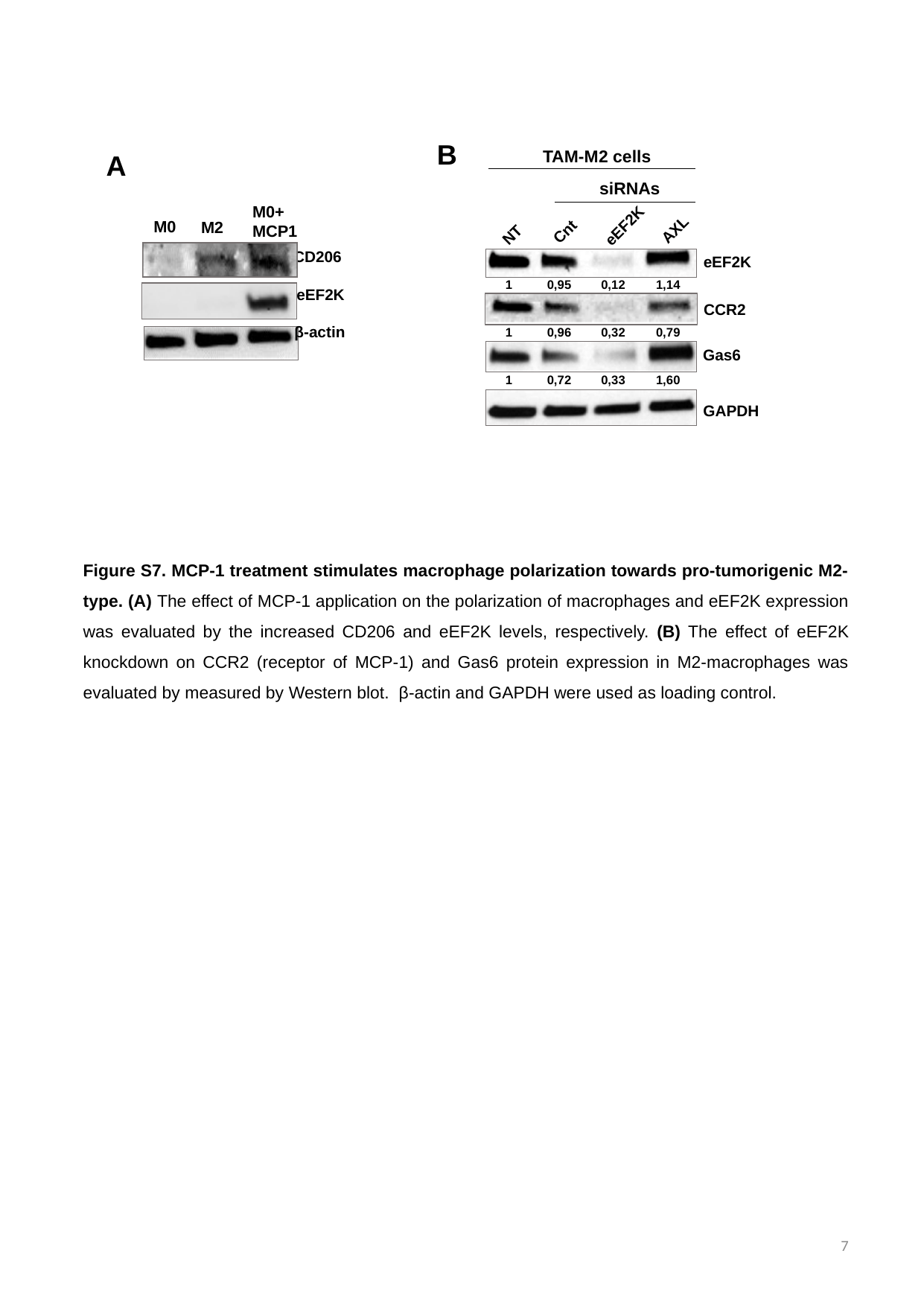

B
TAM-M2 cells
AXL
Cnt
NT
eEF2K
eEF2K
1
0,95
0,12
1,14
CCR2
1
0,96
0,32
0,79
Gas6
1
0,72
0,33
1,60
GAPDH
siRNAs
A
M0+
MCP1
M0
M2
CD206
eEF2K
β-actin
Figure S7. MCP-1 treatment stimulates macrophage polarization towards pro-tumorigenic M2-type. (A) The effect of MCP-1 application on the polarization of macrophages and eEF2K expression was evaluated by the increased CD206 and eEF2K levels, respectively. (B) The effect of eEF2K knockdown on CCR2 (receptor of MCP-1) and Gas6 protein expression in M2-macrophages was evaluated by measured by Western blot. β-actin and GAPDH were used as loading control.
7

## Slide 8
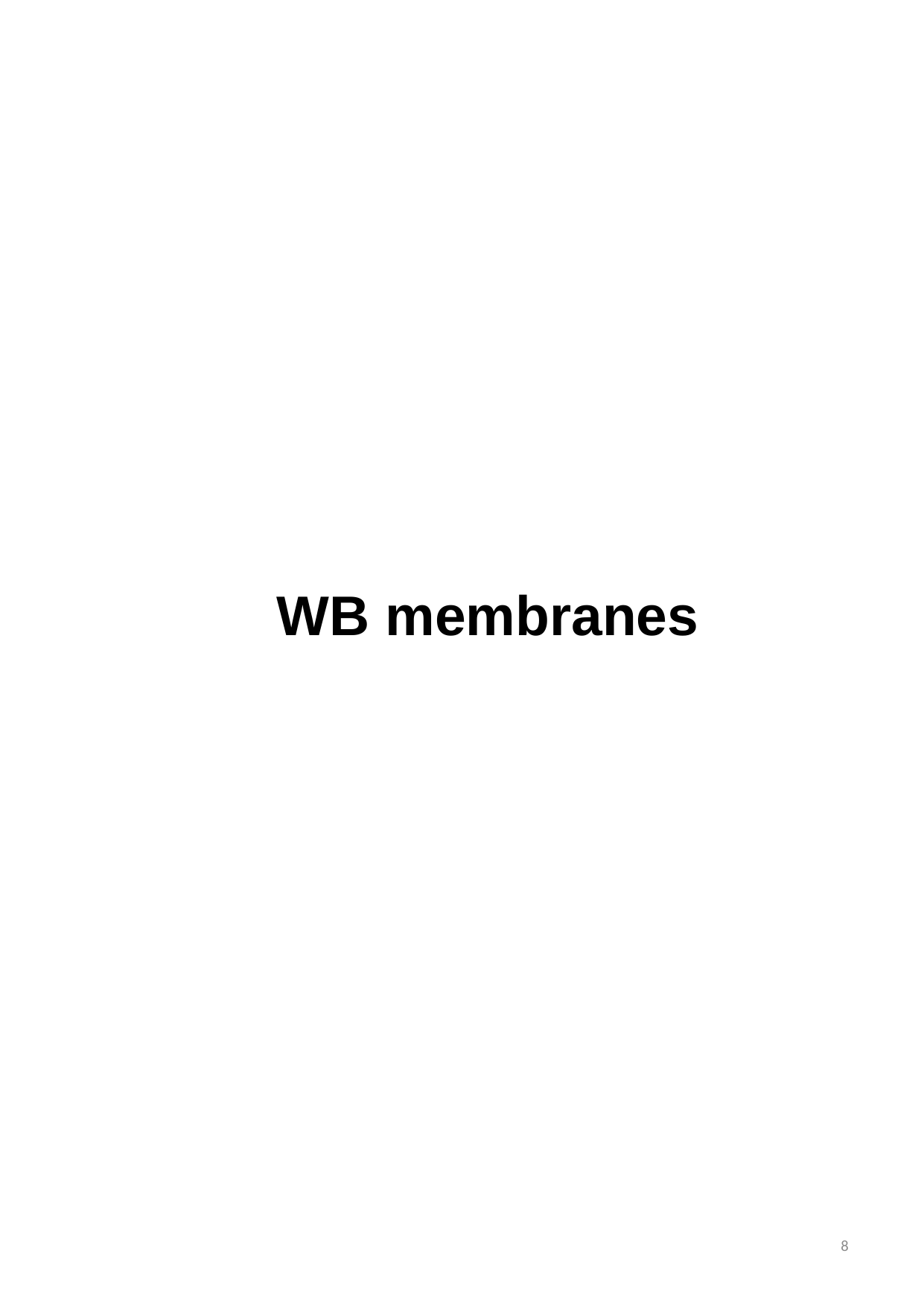

WB membranes
8

## Slide 9
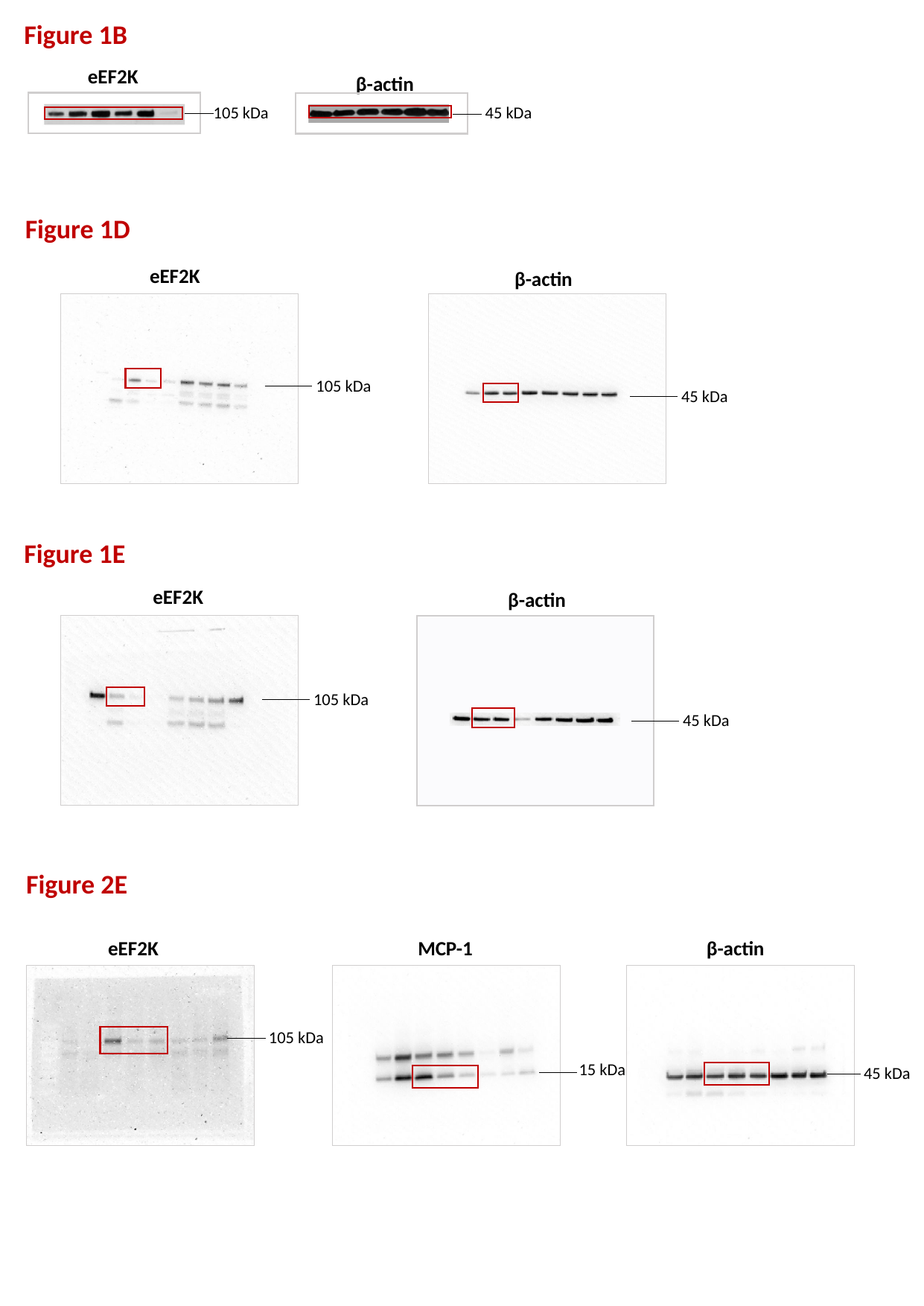

Figure 1B
eEF2K
105 kDa
β-actin
 45 kDa
Figure 1D
eEF2K
β-actin
105 kDa
45 kDa
Figure 1E
eEF2K
β-actin
105 kDa
45 kDa
Figure 2E
eEF2K
MCP-1
β-actin
105 kDa
15 kDa
45 kDa

## Slide 10
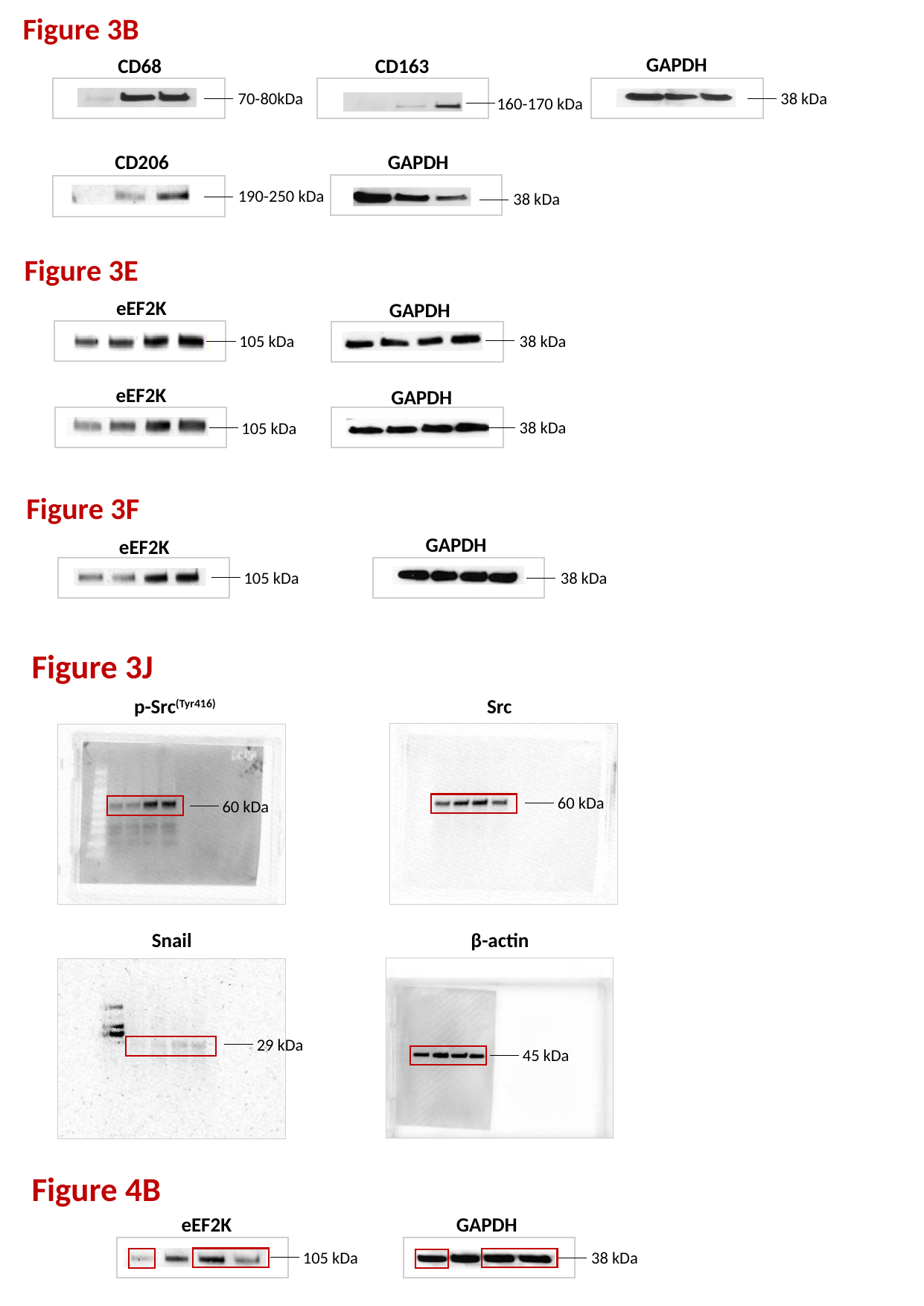

Figure 3B
GAPDH
CD68
CD163
70-80kDa
160-170 kDa
38 kDa
CD206
GAPDH
38 kDa
190-250 kDa
Figure 3E
eEF2K
GAPDH
105 kDa
38 kDa
eEF2K
GAPDH
105 kDa
38 kDa
Figure 3F
GAPDH
eEF2K
105 kDa
38 kDa
Figure 3J
p-Src(Tyr416)
Src
60 kDa
60 kDa
Snail
β-actin
29 kDa
45 kDa
Figure 4B
GAPDH
eEF2K
105 kDa
38 kDa

## Slide 11
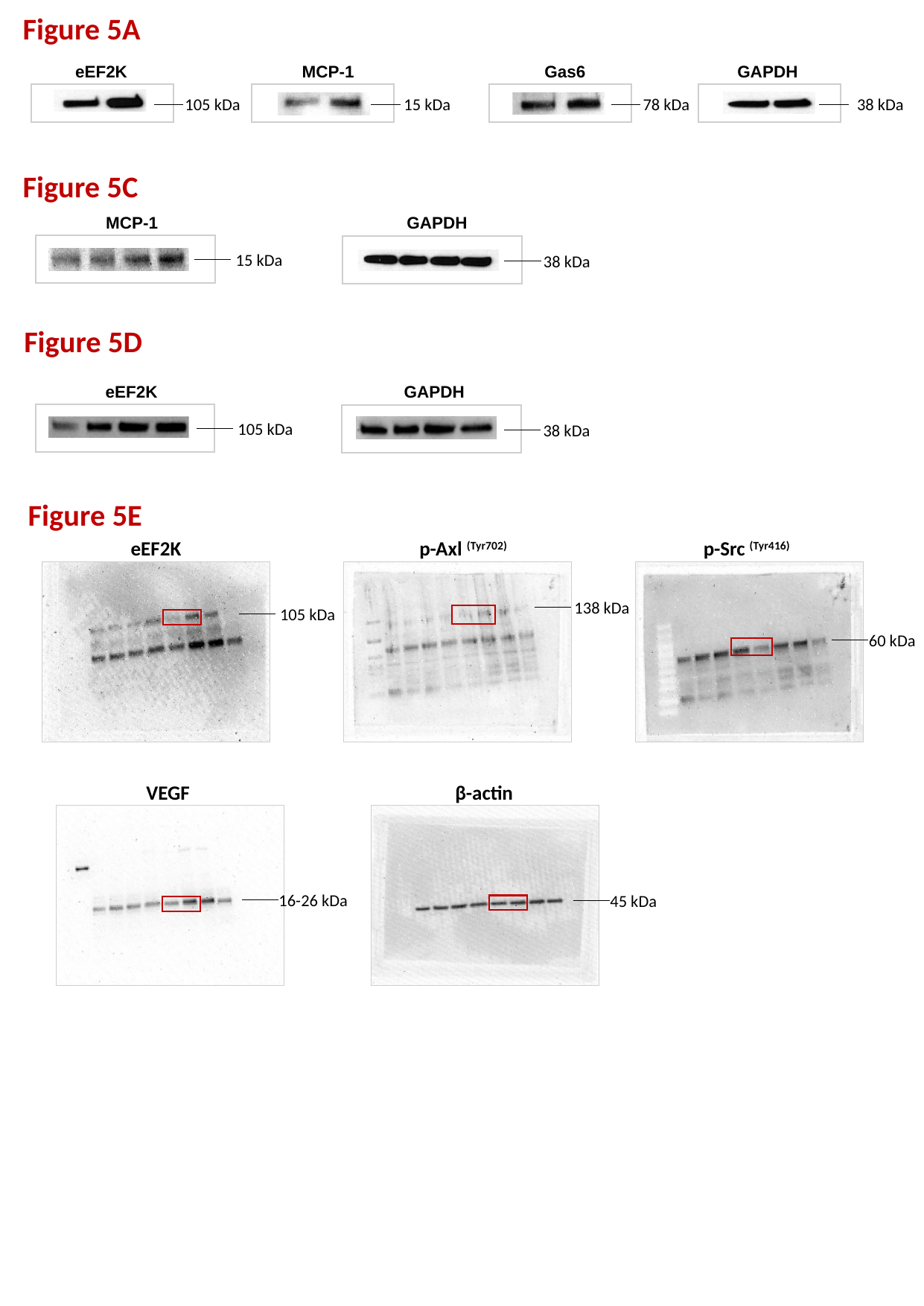

Figure 5A
eEF2K
MCP-1
Gas6
GAPDH
105 kDa
15 kDa
78 kDa
38 kDa
Figure 5C
MCP-1
GAPDH
15 kDa
38 kDa
Figure 5D
eEF2K
GAPDH
105 kDa
38 kDa
Figure 5E
eEF2K
p-Axl (Tyr702)
p-Src (Tyr416)
138 kDa
105 kDa
60 kDa
VEGF
β-actin
16-26 kDa
45 kDa

## Slide 12
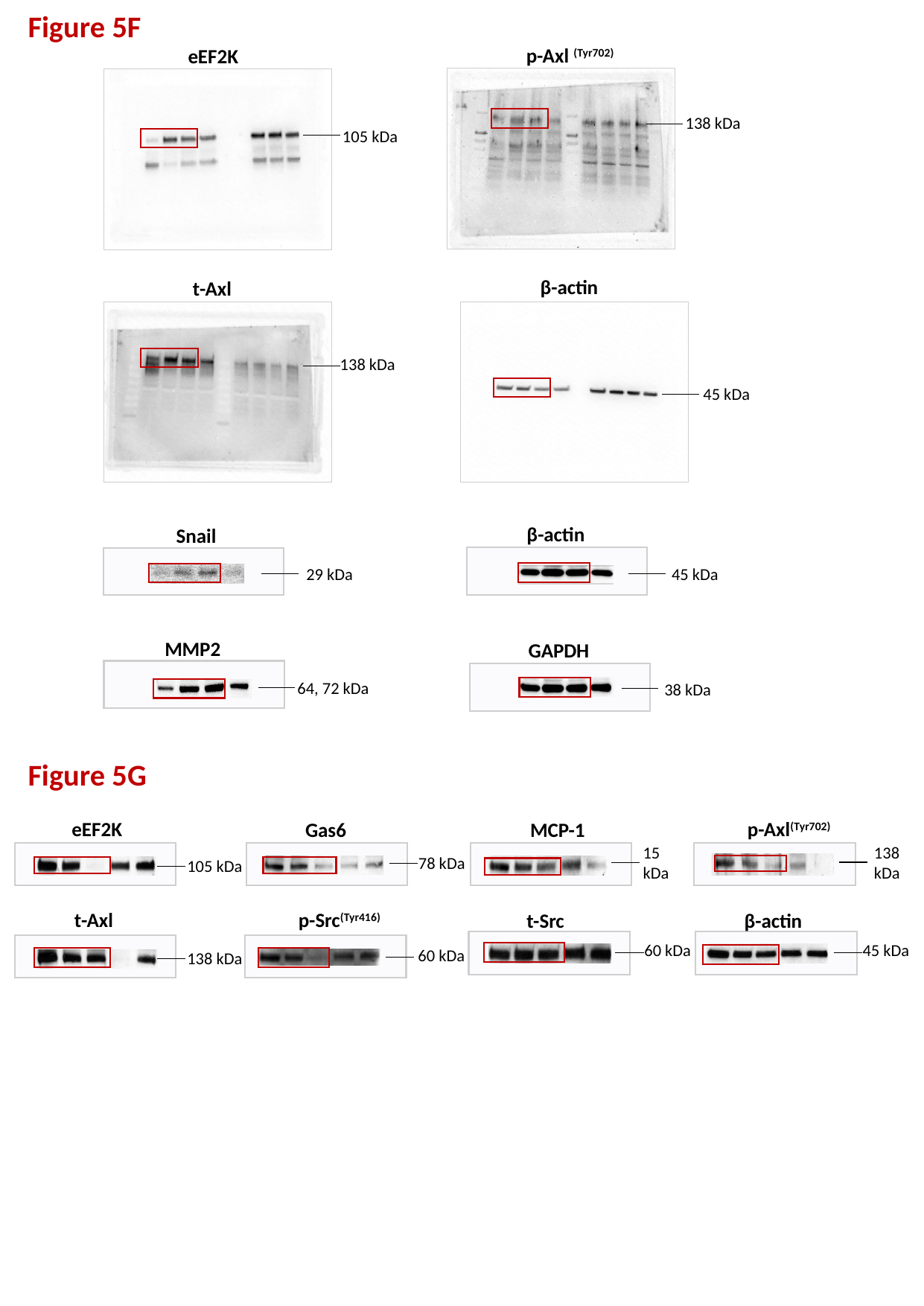

Figure 5F
p-Axl (Tyr702)
eEF2K
138 kDa
105 kDa
β-actin
t-Axl
138 kDa
45 kDa
β-actin
Snail
29 kDa
45 kDa
MMP2
GAPDH
64, 72 kDa
38 kDa
Figure 5G
p-Axl(Tyr702)
eEF2K
MCP-1
Gas6
15
kDa
138
kDa
78 kDa
105 kDa
p-Src(Tyr416)
t-Axl
t-Src
β-actin
45 kDa
60 kDa
60 kDa
138 kDa

## Slide 13
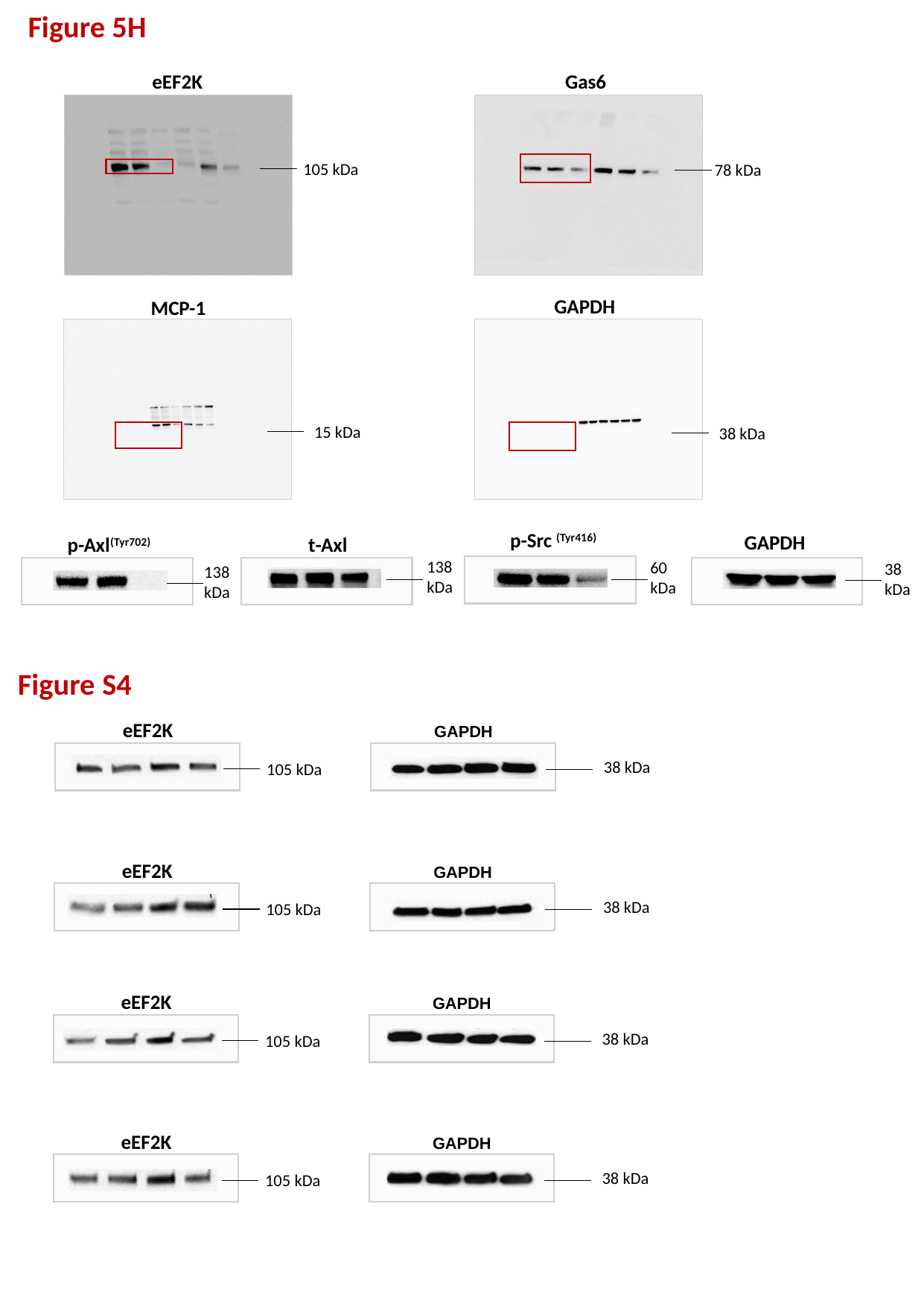

Figure 5H
eEF2K
Gas6
105 kDa
78 kDa
GAPDH
MCP-1
15 kDa
38 kDa
p-Src (Tyr416)
GAPDH
p-Axl(Tyr702)
t-Axl
138
kDa
60
kDa
38
kDa
138
kDa
Figure S4
eEF2K
GAPDH
38 kDa
105 kDa
eEF2K
GAPDH
38 kDa
105 kDa
eEF2K
GAPDH
38 kDa
105 kDa
eEF2K
GAPDH
38 kDa
105 kDa

## Slide 14
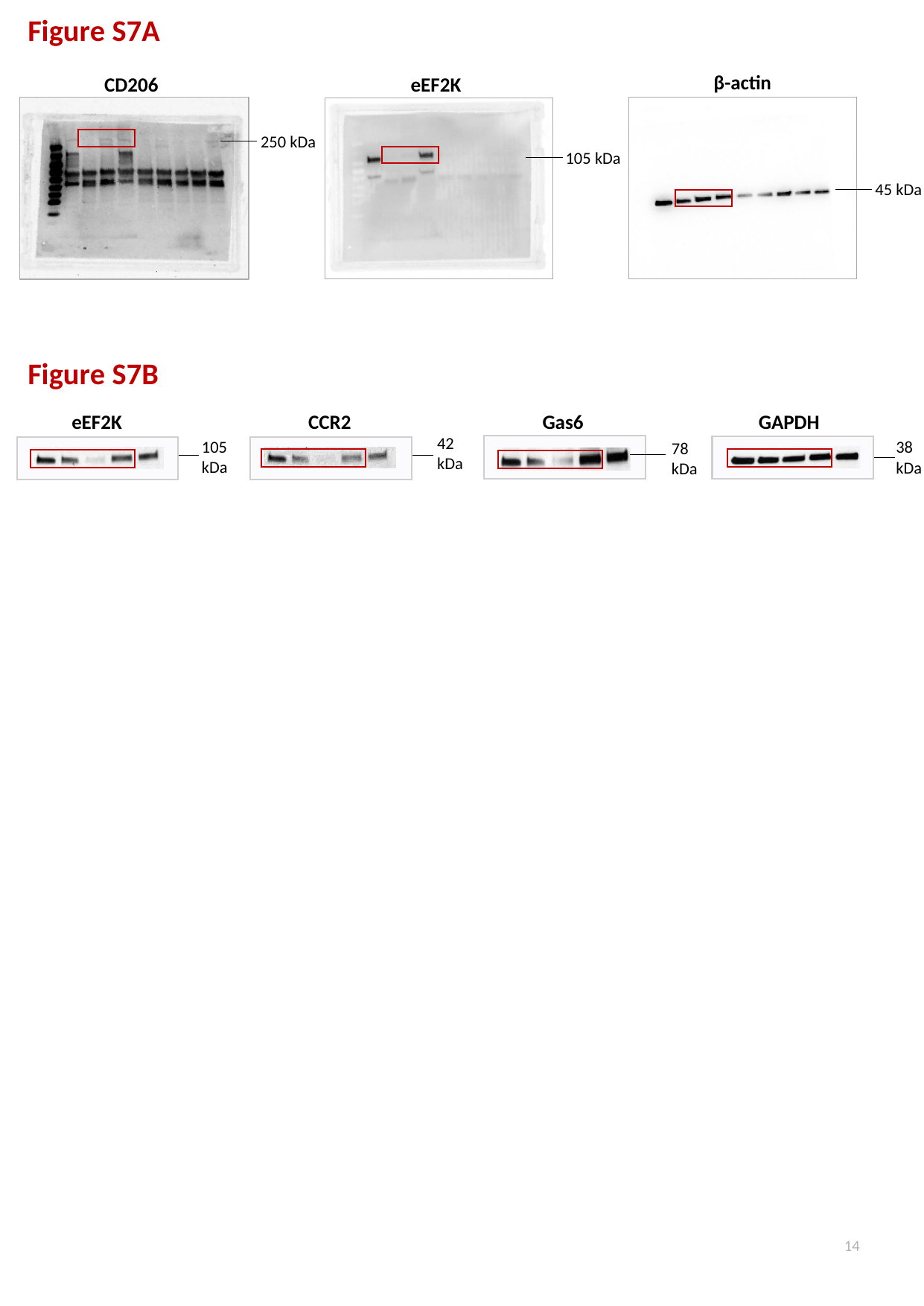

Figure S7A
β-actin
CD206
eEF2K
250 kDa
105 kDa
45 kDa
Figure S7B
eEF2K
CCR2
Gas6
GAPDH
42
kDa
105
kDa
38
kDa
78
kDa
14
