# Supplementary material for: eEF2K is a poor prognostic factor and novel molecular target in pancreatic cancer: regulating tumor growth and progression via the tumor microenvironment
Source: Cell Death Dis. 2025 Jul 7;16(1):501. doi: 10.1038/s41419-025-07803-w (PMC12234705; doi:10.1038/s41419-025-07803-w)
Supplement: Supplementary file 2 — antibody list [file 41419_2025_7803_MOESM2_ESM.pdf]

Cell Signaling Technology, Danvers, MA, USA
eEF2K (CST, #3692)
p-eEF2 (Thr56) (CST, #2331)
Src (CST, #2109S)
p-Src (Tyr416) (CST, #6943)
Snail (CST, #3879)
MMP-2 (CST, #4022S)
CCR2 (CST, #12199)
VEGF (CST, #65373)
p-Axl (Tyr702) (CST, #5724)
Akt (CST, #9272S)
CD163 (CST, #93498)
CD206 (CST, #91992)
Gas 6 (CST, #67202)

Abcam, Cambridge, UK
Gas6 (Abcam, ab136249)
MCP-1 (Abcam, #9669)
CD31 (Abcam, ab28364)

R&D Systems, Minneapolis, MS, USA
Axl (R&D Systems, AF154)

Thermo Fisher, Waltham, MS, USA
Ki-67 (Thermo Fisher, RB-9043-PI)
F4/80 (Thermo Fisher, PA5-32399)

Secondary antibodies (Cell Signaling Technology, Danvers, MA, USA).
HRP-conjugated anti-rabbit (CST #7074)
HRP-conjugated anti-mouse (CST #7076)
